# The antimicrobial drug pyrimethamine inhibits STAT3 transcriptional activity by targeting the enzyme dihydrofolate reductase

**DOI:** 10.1016/j.jbc.2021.101531

**Published:** 2021-12-23

**Authors:** Lisa N. Heppler, Sanaz Attarha, Rosanne Persaud, Jennifer I. Brown, Peng Wang, Boryana Petrova, Isidora Tošić, Foster B. Burton, Yael Flamand, Sarah R. Walker, Jennifer E. Yeh, Roman A. Zubarev, Massimiliano Gaetani, Naama Kanarek, Brent D.G. Page, David A. Frank

**Affiliations:** 1 Department of Medical Oncology, Dana-Farber Cancer Institute, Boston, Massachusetts, USA; 2 Division of Medical Sciences, Harvard University, Boston, Massachusetts, USA; 3 Department of Oncology-Pathology, Karolinska Institute, Stockholm, Sweden; 4 Faculty of Pharmaceutical Sciences, University of British Columbia, Vancouver, Canada; 5 Department of Pathology, Boston Children’s Hospital, Boston, Massachusetts, USA; 6 Harvard Medical School, Boston, Massachusetts, USA; 7 Department of Data Sciences, Dana-Farber-Cancer Institute, Boston, Massachusetts, USA; 8 Division of Physiological Chemistry I, Department of Medical Biochemistry and Biophysics, Karolinska Institute, Stockholm, Sweden; 9 Chemical Proteomics, SciLifeLab, Stockholm, Sweden; 10 Department of Pharmacological & Technological Chemistry, I.M. Sechenov First Moscow State Medical University, Moscow, Russia; 11 The Broad Institute of Harvard and MIT, Cambridge, Massachusetts, USA; 12 Department of Medicine, Brigham and Women’s Hospital, Boston, Massachusetts, USA

**Keywords:** cancer therapy, dihydrofolate reductase, folate metabolism, inhibition mechanism, pyrimethamine, small molecule, STAT3, transcription regulation, BCL6, B-cell lymphoma 6, CETSA, cellular thermal shift assay, CHCHD2, coiled-coil helix–coiled-coil helix domain–containing 2, DHFR, dihydrofolate reductase, DMEM, Dulbecco's modified Eagle's medium, DMSO, dimethyl sulfoxide, dTMP, deoxythymidine monophosphate, FRα, folate receptor α, IP, immunoprecipitation, ITC, isothermal titration calorimetry, JAK2, Janus kinase 2, LGL, large granular lymphocytic, MS, mass spectrometry, NP-40, Nonidet-P40, OSM, oncostatin M, PISA, proteome integral solubility alteration, pol II, RNA polymerase II, pSTAT3, phosphorylated STAT3, qRT, quantitative RT, STAT3, signal transducer and activator of transcription 3, *T*_agg_, aggregation temperature, TBS, Tris-buffered saline, TG101, TG101348, THF, tetrahydrofolate, TMT, tandem mass tag, TPP, thermal proteome profiling, TS, thymidylate synthase

## Abstract

Cancer is often characterized by aberrant gene expression patterns caused by the inappropriate activation of transcription factors. Signal transducer and activator of transcription 3 (STAT3) is a key transcriptional regulator of many protumorigenic processes and is persistently activated in many types of human cancer. However, like many transcription factors, STAT3 has proven difficult to target clinically. To address this unmet clinical need, we previously developed a cell-based assay of STAT3 transcriptional activity and performed an unbiased and high-throughput screen of small molecules known to be biologically active in humans. We identified the antimicrobial drug pyrimethamine as a novel and specific inhibitor of STAT3 transcriptional activity. Here, we show that pyrimethamine does not significantly affect STAT3 phosphorylation, nuclear translocation, or DNA binding at concentrations sufficient to inhibit STAT3 transcriptional activity, suggesting a potentially novel mechanism of inhibition. To identify the direct molecular target of pyrimethamine and further elucidate the mechanism of action, we used a new quantitative proteome profiling approach called proteome integral solubility alteration coupled with a metabolomic analysis. We identified human dihydrofolate reductase as a target of pyrimethamine and demonstrated that the STAT3-inhibitory effects of pyrimethamine are the result of a deficiency in reduced folate downstream of dihydrofolate reductase inhibition, implicating folate metabolism in the regulation of STAT3 transcriptional activity. This study reveals a previously unknown regulatory node of the STAT3 pathway that may be important for the development of novel strategies to treat STAT3-driven cancers.

Cancer is often characterized by aberrant gene expression patterns that alter cellular function. Such alterations are commonly caused by the inappropriate activation of transcription factors. Signal transducer and activator of transcription 3 (STAT3), in particular, is a key transcriptional activator of many protumorigenic processes, including inflammation, proliferation, and survival. STAT3 is persistently phosphorylated and activated in many types of human cancer, including hematologic malignancies and solid tumors (1). Accordingly, STAT3 is a promising therapeutic target for the treatment of cancer.

However, like many transcription factors, STAT3 has proven difficult to target therapeutically (2, 3, 4, 5, 6, 7). To address this unmet clinical need, we performed an unbiased high-throughput screen of the Prestwick collection, a library of 1120 small molecules known to be biologically active in humans, to identify small-molecule inhibitors of STAT3 that could be immediately used in the clinic. We identified the antimicrobial drug pyrimethamine as a novel and specific inhibitor of STAT3 transcriptional activity at concentrations known to be safely achieved in humans (8, 9).

Here, we use a proteome-wide approach coupled with a metabolomic analysis to identify the direct molecular targets of pyrimethamine and further elucidate the mechanism by which pyrimethamine inhibits STAT3 transcriptional activity. As an Food and Drug Administration–approved compound, pyrimethamine is already being tested in the clinic and has shown activity in the treatment of several STAT3-driven malignancies, including chronic lymphocytic leukemia (10), breast cancer, and intermediate-risk to high-risk myelodysplastic syndrome. Therefore, an understanding of the mechanism of action of pyrimethamine may have major clinical implications regarding both the rational design of combination therapies and patient stratification.

## Results

### Pyrimethamine inhibits STAT3 transcriptional activity downstream of DNA binding

**Figure 1:**
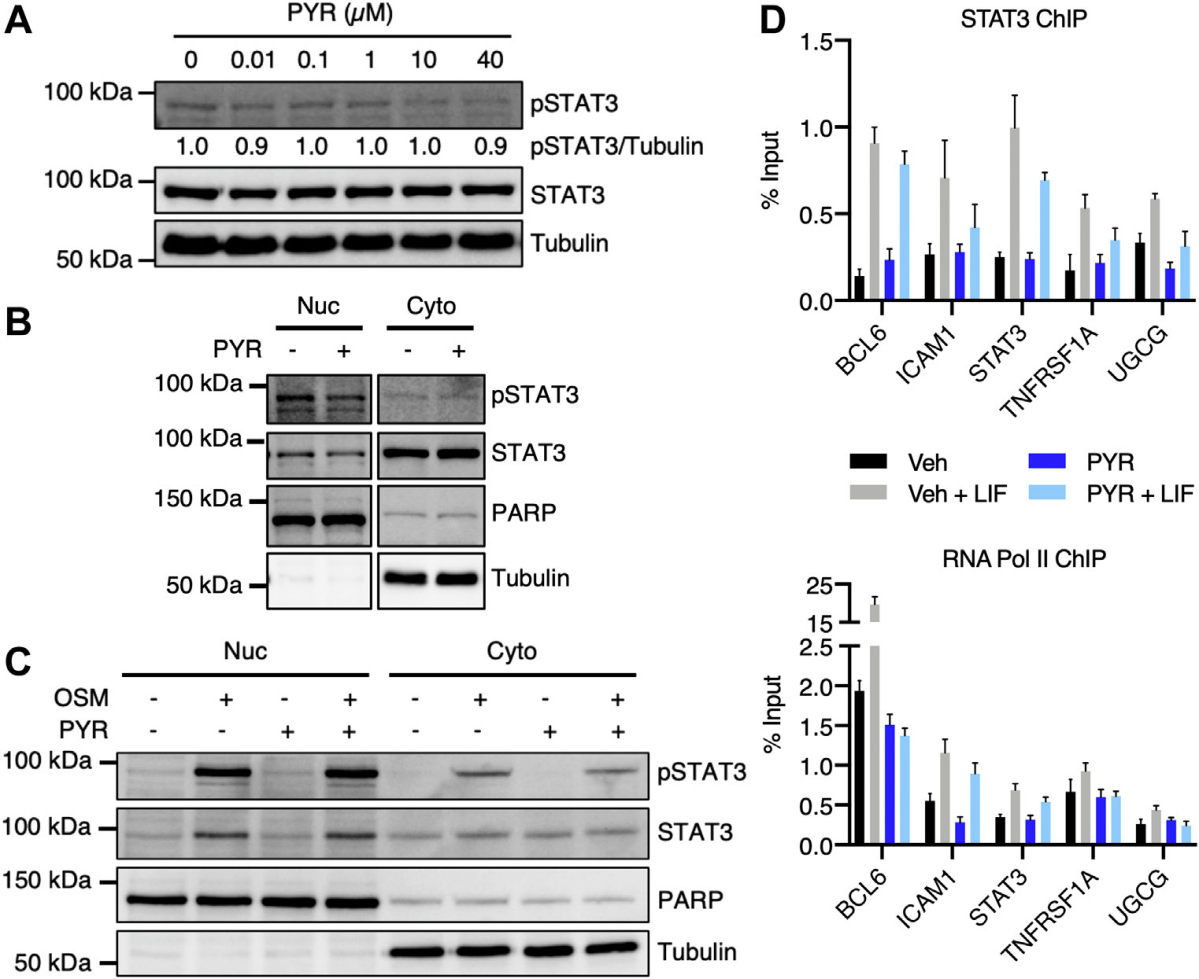
**Pyrimethamine (PYR) inhibits STAT3 transcriptional activity without significantly decreasing STAT3 phosphorylation, nuclear localization, or DNA binding.***A*, MDA-MB-468 cells were treated with PYR for 24 h and analyzed by immunoblotting with the indicated antibodies. pSTAT3 levels were quantified and normalized to tubulin. *B*, MDA-MB-468 cells were treated with 5 μM PYR for 24 h. Nuclear and cytoplasmic fractions were analyzed by immunoblotting with the indicated antibodies. PARP and tubulin were used as nuclear and cytoplasmic markers, respectively. *C*, U3A human fibrosarcoma cells stably expressing STAT3-dependent luciferase were pretreated with 10 μM PYR for 1 h and stimulated with 10 ng/ml oncostatin M (OSM) for 30 min. Nuclear and cytoplasmic fractions were analyzed by immunoblotting with the indicated antibodies. PARP and tubulin were used as nuclear and cytoplasmic markers, respectively. *D*, SKBR3 cells were treated with 10 μM PYR for 1 h, stimulated with 10 ng/ml leukemia inhibitory factor (LIF) for 30 min, and analyzed by chromatin immunoprecipitation with antibodies to STAT3 and RNA pol II (pol II). Binding at the indicated target genes was assessed by quantitative RT–PCR. Data are expressed as percent of input. PARP, poly(ADP-ribose) polymerase; pSTAT3, phosphorylated STAT3; STAT3, signal transducer and activator of transcription 3.

To dissect the mechanism of action of pyrimethamine as a STAT3 inhibitor, we first examined the effect of pyrimethamine on three major steps of the STAT3 signaling pathway, namely STAT3 tyrosine phosphorylation, STAT3 nuclear localization, and STAT3 DNA binding. In MDA-MB-468 cells, a triple-negative breast cancer cell line characterized by constitutive STAT3 phosphorylation, pyrimethamine did not significantly reduce total STAT3 tyrosine phosphorylation (phosphorylated STAT3 [pSTAT3]; Fig. 1*A*) and only led to a slight reduction in nuclear pSTAT3 and total STAT3 (Fig. 1*B*). In addition, in U3A human fibrosarcoma cells (which lack basal STAT3 phosphorylation), pyrimethamine had no effect on oncostatin M (OSM)-stimulated nuclear pSTAT3 or total STAT3 (Fig. 1*C*). Finally, although pyrimethamine had variable effects on leukemia inhibitory factor–stimulated STAT3 and RNA polymerase II (pol II) binding at the five target genes examined, including STAT3 (Fig. 1*D*), the minimal reduction of STAT3 binding at B-cell lymphoma 6 (BCL6), along with the complete loss of pol II recruitment at this site, suggests that pyrimethamine may inhibit STAT3 transcriptional activity by preventing the recruitment of transcriptional regulators, including pol II. It should be noted that, given the variability inherent in chromatin immunoprecipitation (IP), few of the individual changes in binding reached statistical significance. However, the trend in each of the five genes was identical. In the original high-throughput screen that identified pyrimethamine as a STAT3 inhibitor (8), pyrimethamine had no effect on the transcriptional activity of NF-κB or the highly homologous family member STAT5, suggesting that pyrimethamine selectively inhibits STAT3-directed pol II recruitment. Together, these results support pyrimethamine as a novel STAT3 inhibitor that acts downstream of STAT3 tyrosine phosphorylation, nuclear translocation, and DNA binding unlike tyrosine kinase inhibitors and Src homology 2 domain inhibitors.

### Proteome integral solubility alteration identifies dihydrofolate reductase as a direct molecular target of pyrimethamine

To deconvolute the molecular targets of pyrimethamine and further elucidate the mechanism of action of pyrimethamine as a novel STAT3 inhibitor, we used proteome integral solubility alteration (PISA) (11), a proteome-wide profiling approach based on the concept that perturbation agents, such as elevated temperatures, can be used to probe changes in protein solubility induced by small-molecule binding. The underlying hypothesis is that the solubility of a protein bound by a compound of interest will be more or less susceptible to denaturing conditions than the unbound protein. Heat-induced denaturation leads to precipitation in solution, so solubility alteration is also related to a different thermal stability. Thus, the differential protein abundance in the soluble fraction after heat denaturation in treated *versus* untreated cells can be used to directly quantify drug-induced shifts in protein solubility for all proteins identified and quantified across all biological replicates.

Standard thermal proteome profiling (TPP) (12) requires the quantification of the soluble fraction at each temperature point to fit a sigmoidal curve for each protein in the treated and untreated samples to extract differential melting temperatures. TPP data analysis is thus quite extensive and includes several possible sources of error, with a risk of information loss because of poor curve fitting, which limits final depth of the proteomics analysis. In TPP, each biological replicate is associated with high cost and large sample requirements, leading to a limited number of replicates analyzed, reduced throughput, and low statistical power. In contrast, PISA directly measures the sum of the protein amounts in the soluble fraction after solubility perturbation at different perturbation levels. In PISA, *T*, temperature, is used as the agent inducing protein precipitation, whereas other agents are also possible, and the signal corresponds to the integral of a melting curve in TPP, regardless of its shape. As PISA design allows for more biological replicates than TPP, each one with lower sample requirement and costs, the higher statistical significance of protein solubility shifts adds confidence in classification of putative direct molecular targets of the compound of interest.

**Figure 2:**
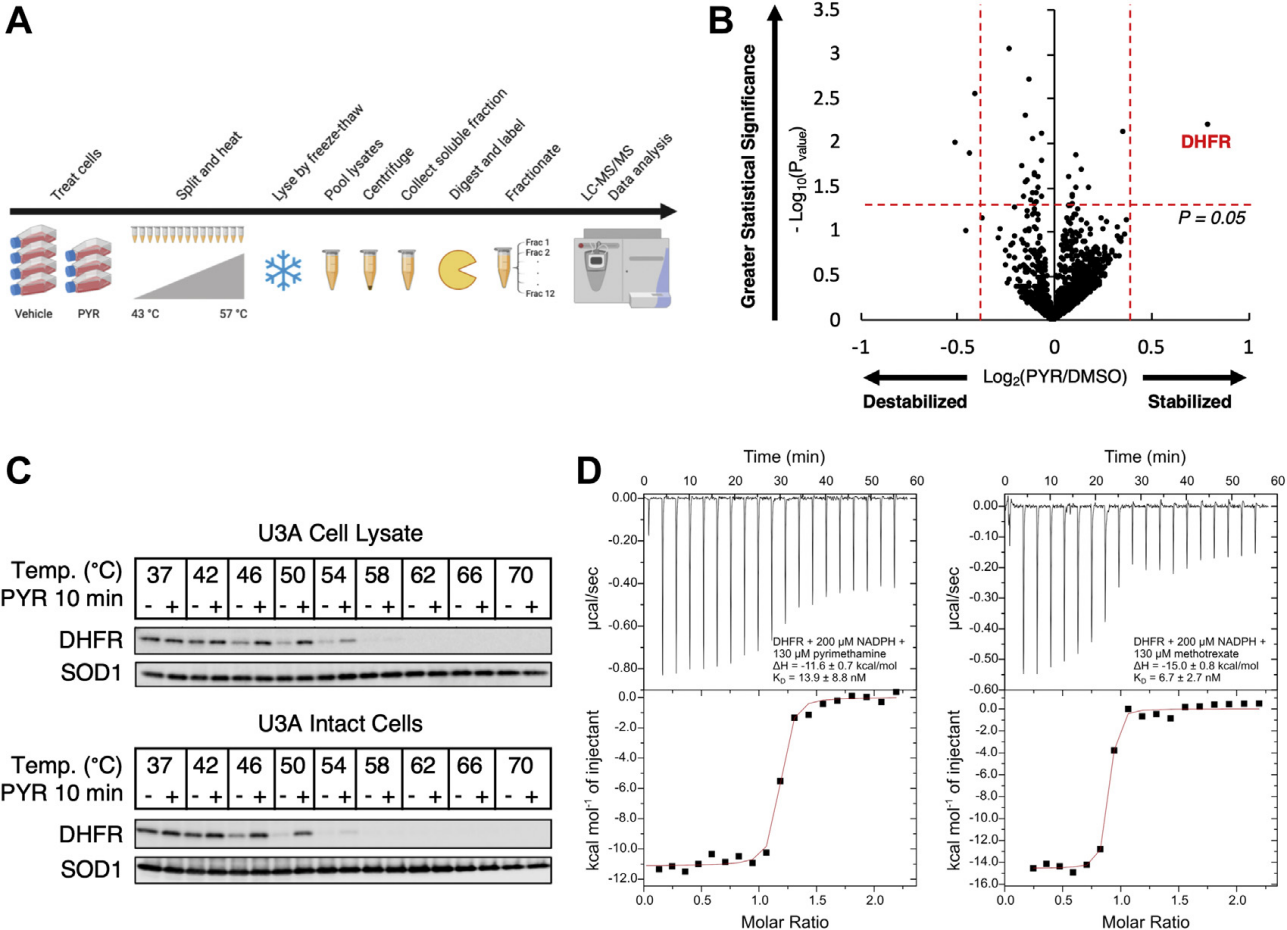
**PISA identifies DHFR as a direct molecular target of pyrimethamine (PYR).***A*, schematic overview of PISA. In brief, each replicate sample was split into 15 equal parts, and each part was heated at a specific temperature. Cells were lysed by freeze–thaw cycles, and lysates were pooled together prior to ultracentrifugation. The soluble fraction was collected, digested, TMT labeled, and analyzed by high-resolution LC–MS/MS. Adapted from Gaetani *et al.*, *J. Proteome Res.*, 2019. Created with BioRender. *B*, U3A cells were treated with 10 μM PYR for 2 h and analyzed by PISA to identify direct molecular targets of PYR. Data presented as a volcano plot. Of the 4462 proteins detected, DHFR was the most stabilized. Proteins with a *p* value less than 0.05 are shown above the *horizontal red dotted line*. Proteins highly stabilized or destabilized by PYR are shown to the far *right* and far *left* of the *vertical red dotted lines*, respectively. *C*, U3A lysate and intact cells were treated with 10 μM PYR for 10 min. Following treatment, samples were heated to the indicated temperatures. Soluble protein levels were analyzed by immunoblotting with the indicated antibodies. SOD1 was used as a thermostable control. *D*, a representative ITC titration curve (*top*) and binding isotherm (*bottom*) resulting from PYR (*left*) or MTX (*right*) titrated into DHFR in the presence of NADPH. Binding isotherms were fit to the one-site binding model to determine characteristics of binding. Inset enthalpy and calculated *K*_*D*_ values are the average and standard deviations of two independent experiments (n = 2). DHFR, dihydrofolate reductase; ITC, isothermal titration calorimetry; MTX, methotrexate; PISA, proteome integral solubility alteration; SOD1, superoxide dismutase 1; TMT, tandem mass tag.

Using this approach, U3A human fibrosarcoma cells stably expressing STAT3-dependent luciferase were treated for 2 h with vehicle or 10 μM pyrimethamine, a concentration routinely achieved in the serum of patients taking this drug for months at a time (Fig. 2*A*). Following drug or vehicle treatment, each biological replicate (four vehicle treated and three pyrimethamine treated) was split into 15 equal parts and heated to temperature points ranging from 43 to 57 °C with 1 °C intervals. After thermal treatment, cells were lysed by freeze–thaw cycles, pooled together, and ultracentrifuged to remove any insoluble proteins. The remaining soluble fractions corresponding to biological replicates were multiplexed in one tandem mass tag (TMT) quantitative proteomics experiment and analyzed by high-resolution LC–MS/MS. Of the 4462 proteins detected, dihydrofolate reductase (DHFR) was the one most stabilized and kept soluble by pyrimethamine (Fig. 2*B*), with a significant 1.7-fold increased abundance in the soluble fraction (Table S1).

To validate the PISA finding that pyrimethamine significantly stabilizes DHFR in solution to heat denaturation, we performed cellular thermal shift assays (CETSAs) in U3A cell lysates and intact cells. In brief, lysates and intact cells were treated with 10 μM pyrimethamine for 10 min and subjected to heat denaturation followed by centrifugation. Protein abundance was measured by immunoblotting and band quantification. In U3A cell lysates, DHFR denatured around 46.3 °C in the absence of drug and 53.2 °C in the presence of pyrimethamine, undergoing a stabilizing shift in aggregation temperature (*T*_agg_) of 6.9 °C (Figs. 2*C* and S1*A*). Since intracellular enzymes and substrates are diluted and decompartmentalized in cell lysates, drug metabolism is less active (13). Thus, this pyrimethamine-mediated shift in *T*_agg_ in U3A cell lysates suggests that pyrimethamine itself is the active compound. Likewise, in intact U3A cells, DHFR experienced a stabilizing shift of 5 °C, moving from 45.6 °C in the absence of drug to 50.6 °C in the presence of pyrimethamine (Figs. 2*C* and S1*A*). Unlike DHFR, another protein that was stabilized by pyrimethamine in the initial PISA screen, coiled-coil helix–coiled-coil helix domain–containing 2 (CHCHD2; Table S1) failed to validate by CETSA (Fig. S1*B*).

To determine the binding affinity of pyrimethamine to DHFR, isothermal titration calorimetry (ITC) experiments were pursued where pyrimethamine (130 μM) was titrated into DHFR (11 μM) in the presence of NADPH (200 μM) and 1.5% dimethyl sulfoxide (DMSO). The resulting isotherm was fit to the one-site model, and a *K*_*D*_ of 13.9 ± 8.8 nM was determined, with Δ*H* of −11.6 ± 0.7 kcal/mol, Δ*S* of −2.8 ± 1 cal/mol/°C, and a stoichiometry of 1.0 ± 0.2 sites (Fig. 2*D*). This was approximately twofold less potent than the known DHFR inhibitor methotrexate, which was assessed for DHFR binding under analogous conditions as a positive control giving a *K*_*D*_ of 6.7 ± 2.7 nM, Δ*H* of −15.0 ± 0.8 kcal/mol, Δ*S* of −13 ± 2 cal/mol/°C, and a stoichiometry of 0.8 ± 0.02 sites. These results demonstrate that the pyrimethamine-mediated stabilizing effect is rapid and specific to DHFR and confirm that DHFR is a direct molecular target of pyrimethamine.

### Pyrimethamine inhibits human DHFR activity

Although pyrimethamine is a known inhibitor of parasitic DHFR, its role as an inhibitor of human DHFR is less clear. Historically, the therapeutic efficacy of pyrimethamine as an antiparasitic compound was attributed to differences in binding between pyrimethamine and either host DHFR or parasitic DHFR, which exists as a bifunctional protein designated DHFR–thymidylate synthase (DHFR–TS) (14). In other words, since human and plasmodium DHFR only share 26% identity by amino acid sequence, it was thought that pyrimethamine had a higher binding affinity for parasitic DHFR, thus providing a clinically beneficial therapeutic window (15). However, more recent studies have shown that despite the low sequence identity, human and parasitic DHFR share similar tertiary structures and demonstrate nearly identical docking scores and binding capacities for pyrimethamine (16). Moreover, *in vitro* activity assays have demonstrated significantly lower differences in binding than those seen *in vivo*, indicating that the selectivity of inhibitors for parasitic DHFR cannot be explained solely by differences in binding affinity (14). Therefore, there remains a need to clarify whether pyrimethamine functionally inhibits DHFR enzymatic activity in mammalian cells.

**Figure 3:**
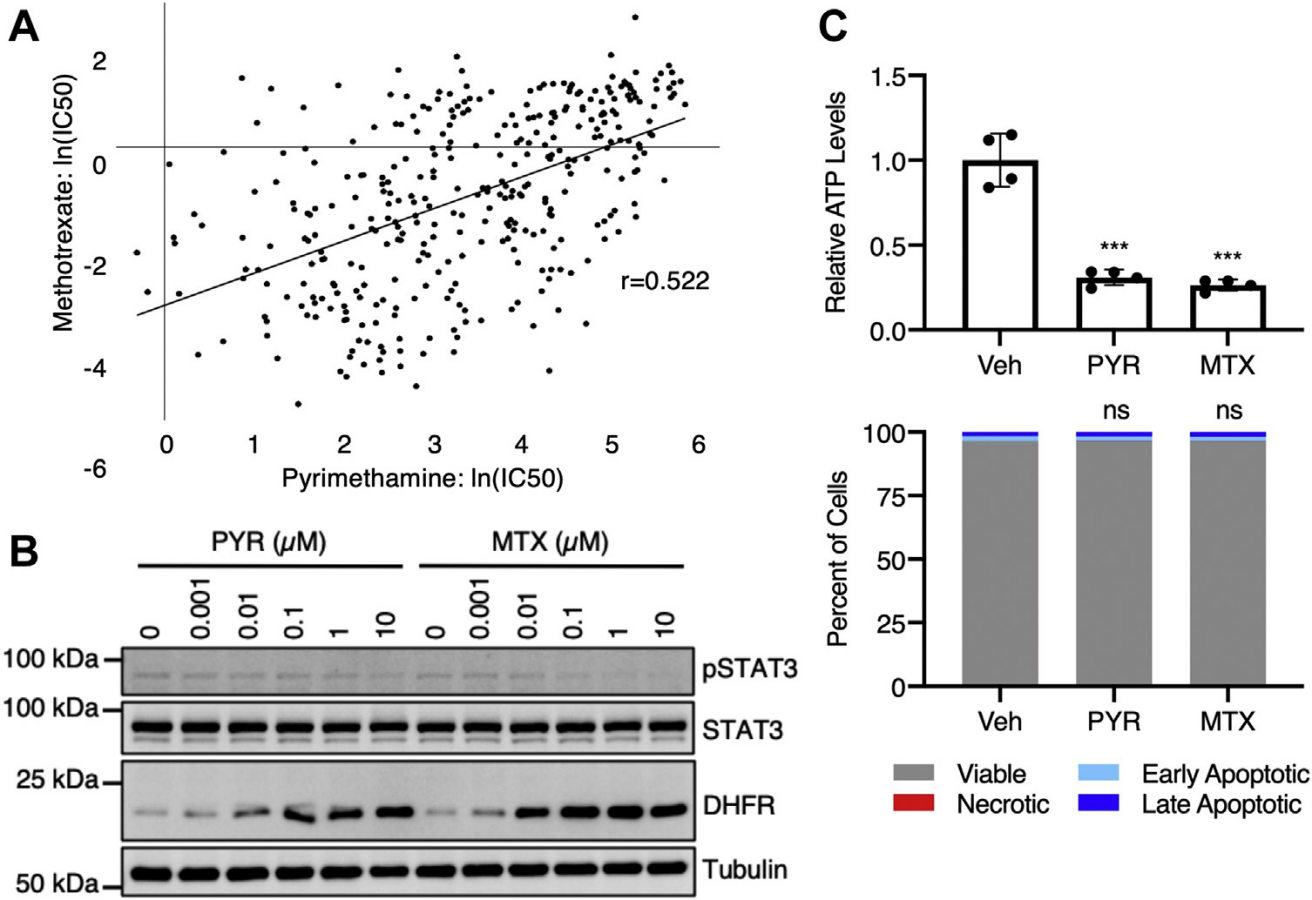
**Pyrimethamine (PYR) inhibits human DHFR activity.***A*, PYR is most similar to methotrexate (MTX), a known inhibitor of human DHFR, in terms of *in vitro* sensitivity in a panel of 345 cancer cell lines (*r* = 0.522; *p* = 1.87E-25). Sensitivity data from the Sanger drug sensitivity IC_50_ dataset accessed *via* the Cancer Dependency Map (Broad Institute). *B*, MDA-MB-468 cells were treated with PYR or MTX for 24 h and analyzed by immunoblotting with the indicated antibodies. *C*, U3A cells were treated with 10 μM PYR or 1 μM MTX for 6 h. ATP levels were measured using CellTiter-Glo and normalized to the vehicle control. In parallel, treated cells were stained with FITC annexin V and propidium iodide (PI) and analyzed by flow cytometry. Statistical comparisons for relative ATP levels were performed using a null of one. Statistical comparisons for flow cytometry were performed using percent of viable and percent of dead (sum of necrotic, early apoptotic, and late apoptotic) between vehicle-treated and drug-treated cells. ns *p* > 0.05, ∗*p* ≤ 0.05, ∗∗*p* ≤ 0.01, and ∗∗∗*p* ≤ 0.001 with two-tailed one-sample Student's *t* test and two-sided Fisher's exact test used in (*C*). DHFR, dihydrofolate reductase; MTX, methotrexate; ns, not significant.

To determine whether pyrimethamine inhibits human DHFR activity, we first used the Cancer Dependency Map Data Explorer (Broad Institute) to probe the Sanger drug sensitivity IC_50_ dataset for correlative relationships (17). We found that pyrimethamine was the most highly correlated with methotrexate, a known inhibitor of human DHFR, in terms of *in vitro* sensitivity across a panel of 345 cancer cell lines (*r* = 0.522; Fig. 3*A*). Of note, the histone deacetylase inhibitor entinostat was the only other drug in the dataset with a Pearson correlation greater than 0.5 (*r* = 0.509). We then examined the effect of pyrimethamine on DHFR protein levels in MDA-MB-468 triple-negative breast cancer cells. Mammalian DHFR is known to inhibit its own translation by binding to the coding region within its own mRNA (18, 19). Accordingly, treatment with methotrexate has been shown to disrupt the interaction between DHFR and its cognate mRNA, releasing the autoinhibitory translational regulation and increasing intracellular levels of DHFR protein (18, 19). Therefore, based on our findings that pyrimethamine interacts with DHFR and mimics the activity of methotrexate *in vitro*, we hypothesized that pyrimethamine would also increase DHFR protein levels. As predicted, pyrimethamine increased DHFR protein levels in a dose-dependent manner (Fig. 3*B*). Interestingly, methotrexate increased DHFR protein levels but at lower concentrations than pyrimethamine, with maximum DHFR levels seen around 0.1 to 1 μM rather than 10 μM (Fig. 3*B*).

Next, we directly tested the inhibitory effect of pyrimethamine on DHFR enzymatic activity *in vitro*. In brief, NADPH absorbance at 340 nm was monitored across increasing doses of pyrimethamine (0.97–2000 μM) or methotrexate (0.0012–2.5 μM), and IC_50_ values were calculated using the 30 min time point. Three independent experiments resulted in a mean IC_50_ of 52 ± 35 μM for pyrimethamine and 0.12 ± 0.07 μM for methotrexate (Table 1 and Fig. S2), corroborating both the ITC and immunoblotting results demonstrating that pyrimethamine is a less potent DHFR inhibitor than methotrexate. Notably, this ∼500-fold difference in potency between pyrimethamine and methotrexate is much higher than what was seen in the ITC results, where pyrimethamine was only approximately twofold less potent. This may be a result of NADPH being required for pyrimethamine binding, but further investigation of this biochemical phenomenon is of interest.

**Table 1:** IC_50_ values of methotrexate and pyrimethamine in DHFR enzymatic assay

Replicate	IC_50_ (M)	
	Methotrexate	Pyrimethamine
RP-21-12	4.93E-08	2.08E-05
RP-21-13	1.14E-07	4.45E-05
RP-21-16	1.91E-07	8.97E-05
Mean ± SD	0.12 ± 0.07 μM	52 ± 35 μM

Finally, we evaluated the effect of pyrimethamine on cellular ATP levels. As a key enzyme in folate metabolism, DHFR supports many biosynthetic processes, including purine and thymidine biosynthesis (20, 21). Accordingly, the inhibition of DHFR with antifolates, such as methotrexate, has been shown to deplete cellular deoxythymidine triphosphate, ATP, and GTP pools (22, 23). Thus, to determine whether pyrimethamine depletes cellular ATP levels, and thus inhibits human DHFR activity, we measured ATP levels following a 6 h treatment with 10 μM pyrimethamine. Consistent with a DHFR-inhibitory effect, pyrimethamine reduced cellular ATP levels by about 75%, which was comparable to that of a 10-fold lower dose of methotrexate (Fig. 3*C*). Since we measured ATP levels using CellTiter-Glo, which is typically used as a proxy for number of viable cells, we also evaluated apoptosis following the same treatment using annexin V and propidium iodide staining followed by flow cytometry. Importantly, nearly 100% of the cells were still viable at the 6 h time point, indicating that the reduction in ATP levels as measured by CellTiter-Glo was not because of a loss of viable cells but rather a drug-specific reduction in cellular ATP. Together, these results demonstrate that pyrimethamine is a human DHFR inhibitor, albeit a less potent inhibitor than methotrexate.

### DHFR loss reduces STAT3 transcriptional activity

**Figure 4:**
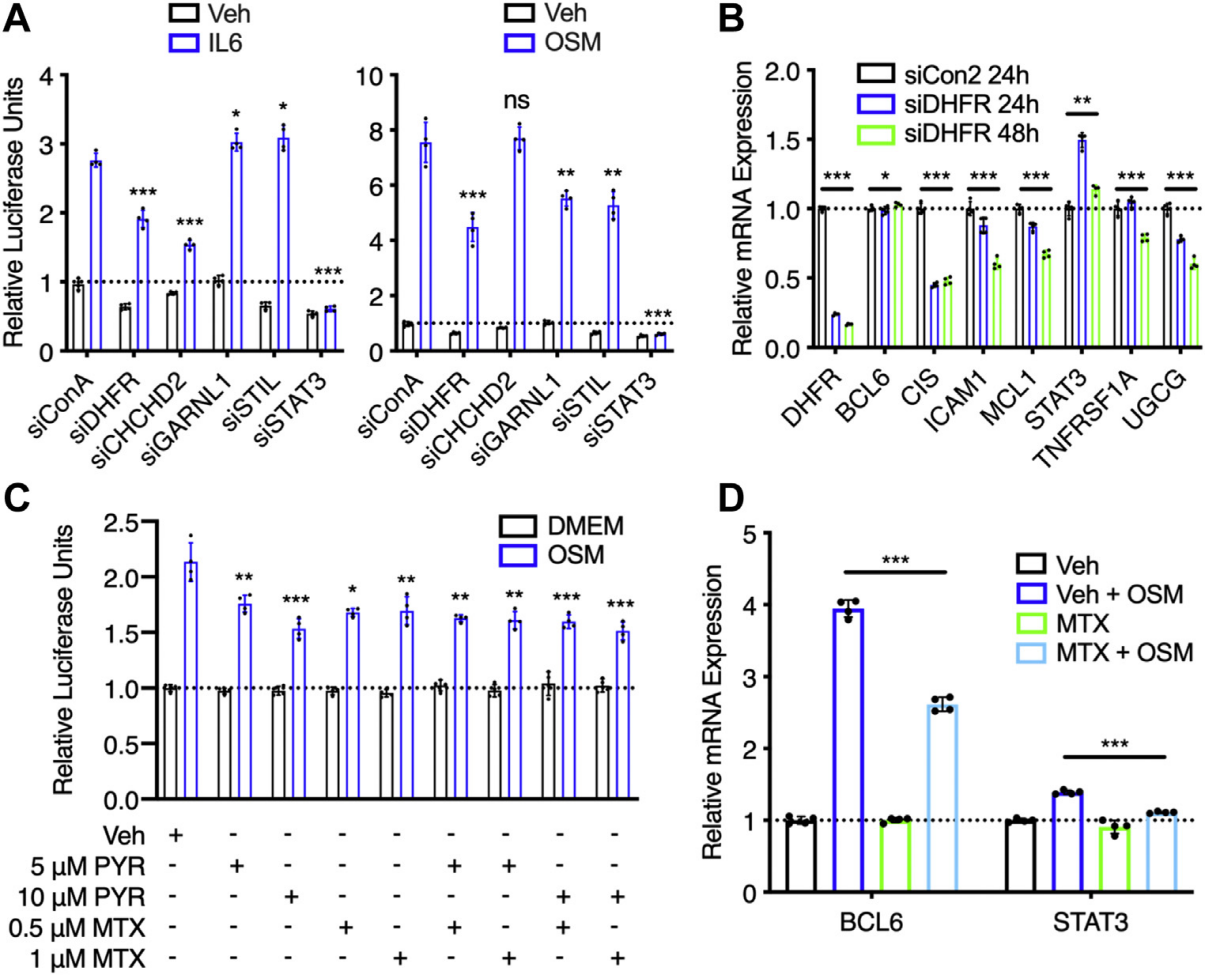
**Inhibition of DHFR decreases cytokine-stimulated STAT3 transcriptional activity and STAT3 target gene expression.***A*, U3A cells were transfected with siRNA targeting DHFR and three other hits from the PISA screen, namely CHCHD2, STIL, and GARNL1. siRNA targeting STAT3 was used as a positive control. About 48 h later, the cells were stimulated with 10 ng/ml interleukin-6 (IL-6) or 10 ng/ml OSM for 6 h. Luciferase was measured and normalized to siConA DMEM. Statistical comparisons were performed between the indicated mean values and that of siConA IL-6/OSM. *B*, MDA-MB-468 cells were transfected with siRNA targeting DHFR and analyzed by quantitative RT–PCR. mRNA levels were normalized to GAPDH and nontargeting siCon2. *C*, U3A cells were pretreated with pyrimethamine (PYR) and/or methotrexate (MTX) for 1 h and stimulated with 10 ng/ml OSM for 5 h. Luciferase was measured and normalized to the unstimulated vehicle control. Statistical comparisons were performed between the indicated mean values and that of DMSO OSM. *D*, U3A cells were pretreated with 1 μM MTX for 1 h, stimulated with 10 ng/ml OSM for 90 min, and analyzed by quantitative RT–PCR. mRNA levels were normalized to GAPDH and unstimulated vehicle control. ns *p* > 0.05, ∗*p* ≤ 0.05, ∗∗*p* ≤ 0.01, and ∗∗∗*p* ≤ 0.001 with two-tailed unpaired Student's *t* test used in (*A*), two-tailed one-sample Student's *t* test used in (*B*), and two-tailed unpaired Student's *t* test (with or without Welch's correction depending on *F* test) used in (*C*) and (*D*). CHCHD2, coiled-coil helix–coiled-coil helix domain–containing 2; DHFR, dihydrofolate reductase; DMEM, Dulbecco's modified Eagle's medium; DMSO, dimethyl sulfoxide; GARNL1, GTPase-activating Rap/Ran-GAP domain–like 1; ns, not significant; OSM, oncostatin M; PISA, proteome integral solubility alteration; STAT3, Signal transducer and activator of transcription 3; STIL, SCL/TAL1 interrupting locus.

Based on our findings, we proposed two hypothetical models to explain the mechanism of action of pyrimethamine as a STAT3 inhibitor. In the first model, DHFR acts as a transcriptional activator of STAT3. Accordingly, pyrimethamine inhibits STAT3 transcriptional activity by inhibiting the enzymatic activity of DHFR. In the second model, DHFR acts as a transcriptional repressor of STAT3. Therefore, pyrimethamine inhibits STAT3 transcriptional activity by increasing DHFR protein levels. To differentiate between these two models, we tested the effect of siRNA-mediated knockdown of DHFR on STAT3 transcriptional activity. If DHFR acts as a transcriptional activator, as presented in the first model, loss of DHFR should reduce cytokine-stimulated STAT3 activity. We first used U3A human fibrosarcoma cells stably expressing STAT3-dependent luciferase to determine the effect of knockdown on STAT3 luciferase activity. STAT3-targeting siRNA was used as a positive control. Of the four candidates identified by PISA that were tested (Tables S1 and S2), DHFR knockdown was the only one that significantly reduced both OSM-stimulated and interleukin 6-stimulated STAT3 activity (Fig. 4*A*). Of note, neither basal nor induced STAT3 phosphorylation was significantly affected by DHFR knockdown (Fig. S3). Next, we used MDA-MB-468 cells characterized by constitutive STAT3 activation to test the effect of DHFR knockdown on STAT3 target gene expression. Consistent with the STAT3 luciferase data, DHFR knockdown reduced the expression of a panel of STAT3 target genes, including cytokine-inducible Src homology 2–containing protein, intercellular adhesion molecule 1, myeloid cell leukemia 1, tumor necrosis factor receptor superfamily member 1A, and UDP-glucose ceramide glucosyltransferase (Fig. 4*B*). It should be noted that DHFR knockdown did not decrease expression of some target genes, including BCL6 and STAT3. While effects on a model promoter, such as the luciferase reporter construct, are of large magnitude and generally reproducible across cell types, endogenous genes, which are regulated by a cohort of basal and inducible transcription factors, show lower magnitude and cell context–dependent effects. Together, these results support the model in which DHFR acts as a transcriptional activator of STAT3, raising the possibility that pyrimethamine inhibits STAT3 transcriptional activity, at least in part, by inhibiting DHFR.

### Methotrexate inhibits STAT3 transcriptional activity

Based on the model that pyrimethamine reduces STAT3 transcriptional activity by inhibiting DHFR, we hypothesized that other DHFR inhibitors, specifically methotrexate, would also inhibit STAT3 transcriptional activity. To test this hypothesis, we first evaluated the effect of methotrexate on cytokine-stimulated STAT3 luciferase activity. In brief, U3A cells were pretreated with methotrexate for 1 h and then stimulated with cytokine for 5 h. STAT3-dependent luciferase activity was then used as a measure of STAT3 transcriptional activity. As hypothesized, methotrexate decreased OSM-stimulated STAT3 activity at both 0.5 and 1 μM (Fig. 4*C*), indicating that methotrexate is a more potent STAT3 inhibitor in addition to being a more potent DHFR inhibitor. We also tested the effect of methotrexate on OSM-stimulated STAT3 target gene expression in the same U3A cell line. In accordance with the luciferase data, methotrexate significantly reduced the OSM-stimulated expression of BCL6 and STAT3, two representative STAT3 target genes (Fig. 4*D*).

In addition, we evaluated the combination of pyrimethamine and methotrexate on cytokine-stimulated STAT3 activity. The combination showed no additional inhibitory effect compared with the single agents (Fig. 4*C*), suggesting that both pyrimethamine and methotrexate reduce STAT3 transcriptional activity *via* the same molecular target, namely DHFR. Overall, the ability of both pyrimethamine and methotrexate to inhibit STAT3 activity implies that there is a functional relationship between DHFR enzymatic activity, folate metabolism, and STAT3 transcriptional activity.

### Folinic acid and thymidine rescue the STAT3-inhibitory effects of pyrimethamine

**Figure 5:**
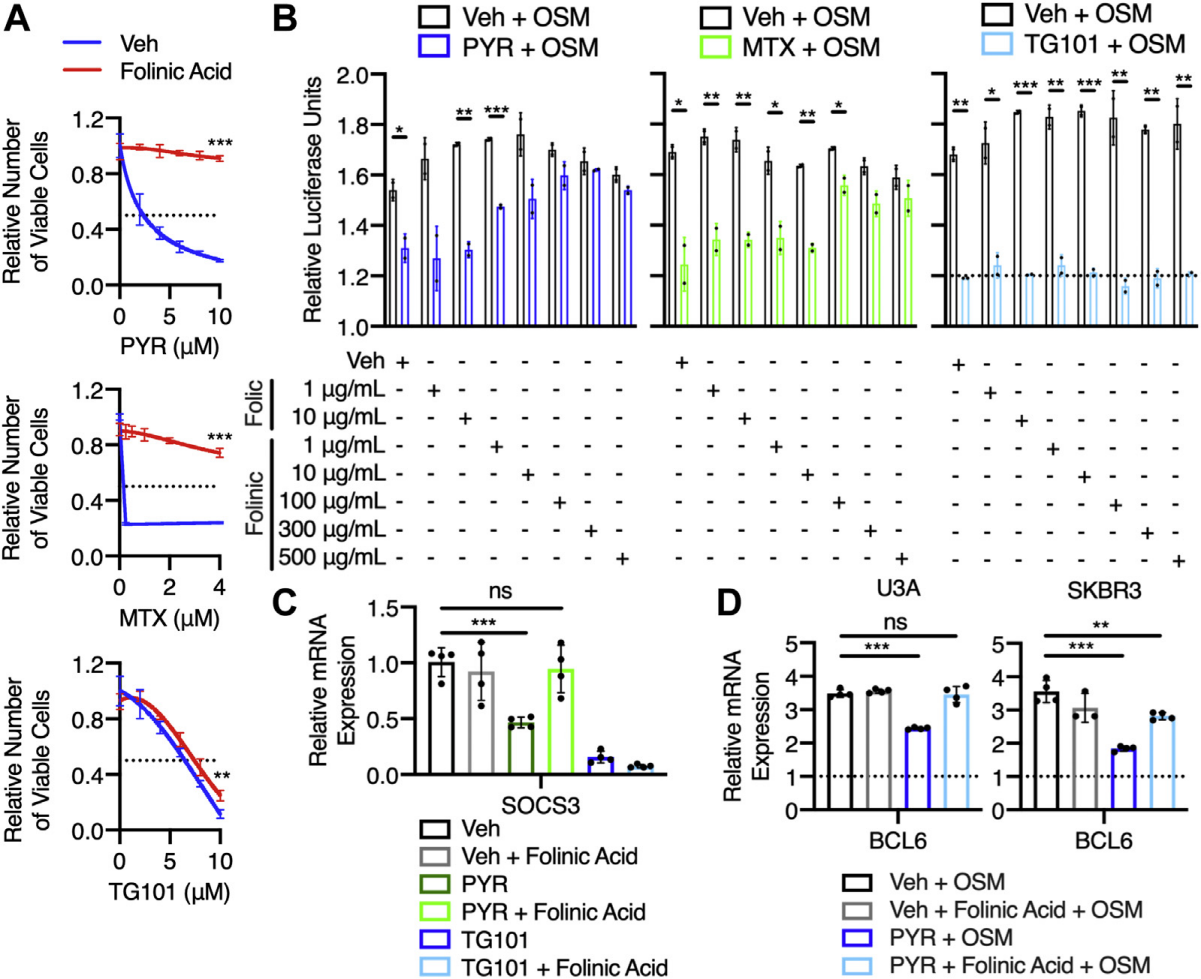
**Folinic acid rescues the STAT3-inhibitory effects of pyrimethamine (PYR).***A*, MDA-MB-468 cells were treated with 300 μg/ml folinic acid and either PYR, methotrexate (MTX), or TG101348 (TG101), a JAK2 inhibitor, for 48 h. The relative number of viable cells was measured using Cell-Titer-Glo and normalized to the respective vehicle control. Statistical comparisons were performed between mean values at the maximum concentration. *B*, U3A cells were pretreated with folic acid or folinic acid, as well as 10 μM PYR, 1 μM MTX, or 10 μM TG101, for 1 h and stimulated with 10 ng/ml oncostatin M (OSM) for 5 h. Luciferase was measured and normalized to the unstimulated vehicle control. Relative luciferase units are presented as fold change over unstimulated. *C*, MDA-MB-468 cells were treated with 300 μg/ml folinic acid and either 10 μM PYR or 10 μM TG101 for 24 h and analyzed by quantitative RT–PCR. mRNA levels were normalized to GAPDH and vehicle control. *D*, U3A and SKBR3 cells were pretreated with 300 μg/ml folinic acid and 10 μM PYR for 1 h, stimulated with 10 ng/ml OSM for 90 and 60 min, respectively, and analyzed by quantitative RT–PCR. mRNA levels were normalized to GAPDH and unstimulated vehicle control. Relative mRNA expression presented as fold change over unstimulated. ns *p* > 0.05, ∗*p* ≤ 0.05, ∗∗*p* ≤ 0.01, and ∗∗∗*p* ≤ 0.001 with two-tailed unpaired Student's *t* test (with or without Welch's correction depending on *F* test) used in (*A*), (*B*), and (*D*) and two-tailed one-sample Student's *t* test used in (*C*). JAK2, Janus kinase 2; ns, not significant; STAT3, signal transducer and activator of transcription 3.

To examine the link between folate metabolism and STAT3 transcriptional activity, we asked whether folinic acid, a reduced form of folate that enters the metabolic pathway downstream of DHFR, could rescue the STAT3-inhibitory effects of pyrimethamine. We specifically hypothesized that folinic acid would prevent pyrimethamine-mediated loss of viability and loss of STAT3 transcriptional activity by bypassing DHFR and restoring the folate-dependent processes required for STAT3 transcriptional activity. To test this hypothesis, we first evaluated the effect of folinic acid on the number of viable MDA-MB-468 cells following a 48 h treatment with pyrimethamine, methotrexate, or the small molecule Janus kinase 2 (JAK2) inhibitor TG101348 (TG101). Since MDA-MB-468 cells are dependent on STAT3 activity for survival, loss of viability can reflect STAT3 inhibition. Folinic acid almost completely prevented pyrimethamine-dependent and methotrexate-dependent decreases in the number of viable cells, while having very little effect on TG101-dependent loss (Fig. 5*A*).

To build on these results, we tested the effect of both folinic acid and folic acid (which would not relieve the block in reduced folate production) on the ability of pyrimethamine to inhibit OSM-stimulated STAT3 activity. Because of solubility limitations, we were only able to test a maximum folic acid concentration of 10 μg/ml; although, this was still 2.5-fold higher than the baseline concentration of folic acid in the culture media, it does limit the ability to determine the effects of folic acid. At the tested concentrations, folinic acid, but not folic acid, significantly reduced the ability of pyrimethamine to inhibit STAT3 transcriptional activity and restored STAT3-dependent luciferase in a dose-dependent manner (Fig. 5*B*). Folinic acid was also able to rescue the effect of methotrexate on STAT3-dependent luciferase but had no effect on TG101-mediated loss, again indicating that pyrimethamine and methotrexate share a STAT3-inhibitory mechanism that is dependent on DHFR enzymatic activity.

To further validate that folinic acid can rescue the STAT3-inhibitory effects of pyrimethamine, we examined the ability of folinic acid to rescue pyrimethamine-mediated loss of STAT3 target gene expression in three cell lines. First, we treated MDA-MB-468 cells with folinic acid plus either pyrimethamine or TG101 for 24 h and analyzed by quantitative RT–PCR (qRT–PCR) expression of SOCS3, a target gene that is highly responsive to STAT3 transcriptional activity (24). As expected, both pyrimethamine and TG101 significantly reduced SOCS3 expression in the absence of folinic acid (Fig. 5*C*). However, in the presence of folinic acid, TG101 still reduced SOCS3 expression, whereas pyrimethamine had no effect. Next, we pretreated both U3A cells and SKBR3 human mammary carcinoma cells (both of which lack basal STAT3 phosphorylation) with folinic acid and pyrimethamine for 1 h and then stimulated with OSM to activate STAT3. Although pyrimethamine on its own significantly reduced OSM-stimulated BCL6 expression in both cell lines, as expected, the addition of folinic acid completely prevented pyrimethamine-mediated loss of OSM-stimulated BCL6 expression in U3A cells and partially prevented the loss in SKBR3 cells (Fig. 5*D*).

**Figure 6:**
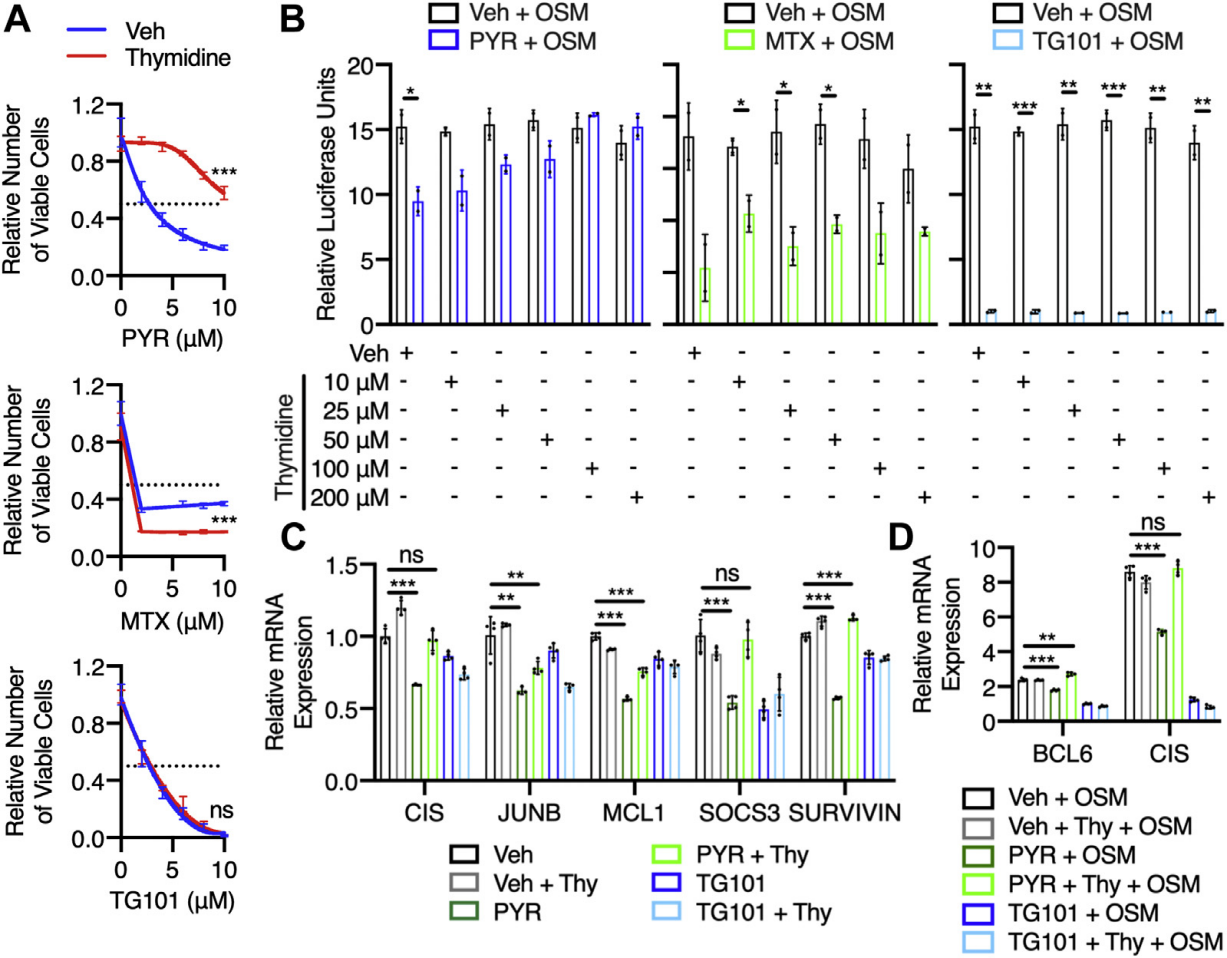
**Thymidine rescues the STAT3-inhibitory effects of pyrimethamine (PYR).***A*, MDA-MB-468 cells were treated with 50 μM thymidine and either PYR, methotrexate (MTX), or TG101348 (TG101), a JAK2 inhibitor, for 48 h. The relative number of viable cells was measured using Cell-Titer-Glo and normalized to the respective vehicle control. Statistical comparisons were performed between mean values at the maximum concentration. *B*, U3A cells were pretreated with thymidine, as well as 10 μM PYR, 1 μM MTX, or 10 μM TG101, for 1 h and stimulated with 10 ng/ml oncostatin M (OSM) for 5 h. Luciferase was measured and normalized to the unstimulated vehicle control. Relative luciferase units are presented as fold change over unstimulated. *C*, MDA-MB-468 cells were treated with 50 μM thymidine and either 10 μM PYR or 1 μM TG101 for 24 h and analyzed by quantitative RT–PCR. mRNA levels were normalized to GAPDH and vehicle control. *D*, U3A cells were pretreated with 50 μM thymidine and either 10 μM PYR or 10 μM TG101, stimulated with 10 ng/ml OSM for 1 h, and analyzed by quantitative RT–PCR. mRNA levels were normalized to GAPDH and unstimulated vehicle control. Relative mRNA expression is presented as fold change over unstimulated. ns *p* > 0.05, ∗*p* ≤ 0.05, ∗∗*p* ≤ 0.01, and ∗∗∗*p* ≤ 0.001 with two-tailed unpaired Student's *t* test (with or without Welch's correction depending on *F* test) used in (*A*), (*B*), and (*D*), and two-tailed one-sample Student's *t* test used in (*C*). JAK2, Janus kinase 2; ns, not significant; STAT3, signal transducer and activator of transcription 3.

Moving downstream of folinic acid, and potentially closer to the folate-mediated process regulating STAT3 transcriptional activity, we evaluated whether thymidine could rescue the STAT3-inhibitory effects of pyrimethamine. Similar to folinic acid, the addition of thymidine reduced the ability of pyrimethamine to inhibit STAT3 transcriptional activity, restoring the number of viable cells (Fig. 6*A*), STAT3-dependent luciferase activity (Fig. 6*B*), and STAT3 target gene expression (Fig. 6, *C* and *D*). However, thymidine was unable to rescue methotrexate-mediated loss of STAT3 activity (Fig. 6, *A* and *B*), suggesting at least a slight difference in the mechanisms of action of pyrimethamine and methotrexate as STAT3 inhibitors. One possible explanation could be related to methotrexate also targeting TS (25) after its being metabolized in cells, as it appears from comparison of PISA data obtained from cells and cell lysates (11). This feature is specific for methotrexate, as no engagement of TS was detected by pyrimethamine. Since thymidine rescues STAT3 function and viability in pyrimethamine-treated cells but not methotrexate-treated cells, this suggests that the alterations in viability seen under these conditions relate to the inhibition of STAT3 and not other effects mediated by DHFR inhibition.

### Pyrimethamine inhibits purine and pyrimidine synthesis in STAT3-driven cancer cells

**Figure 7:**
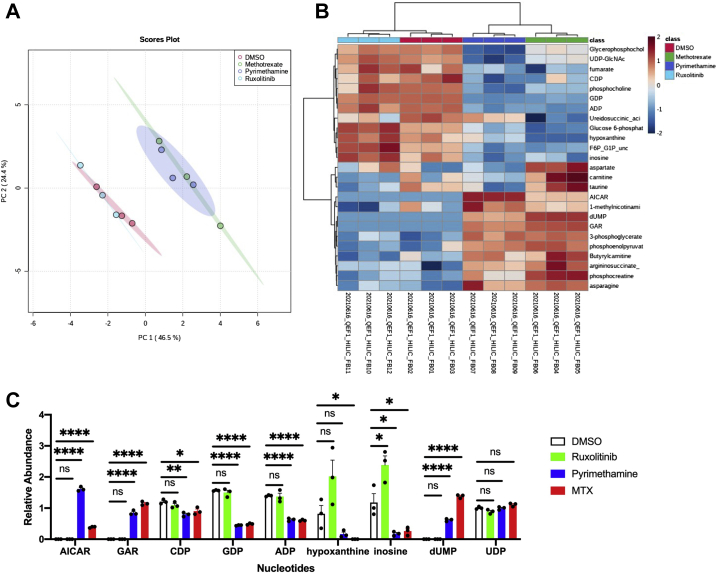
**Metabolite profiling suggests that pyrimethamine is a DHFR inhibitor.***A*, global PCA analysis of polar metabolite detection in MDA-MB-468 cancer cells treated for 24 h with DMSO, 0.05 μM methotrexate, 10 μM pyrimethamine, and 1 μM ruxolitinib. Each replicate represents extracted metabolites from one million cells per condition. *B*, top 25 differentially detected metabolites from (*A*). *C*, detected levels of nucleotides in the treated cells from (*A*). Mean and standard deviation of three technical replicates are presented; statistical significance was determined using multiple *t* test and nonparametric analysis. ns *p* > 0.05, ∗*p* ≤ 0.05, ∗∗*p* ≤ 0.01, and ∗∗∗*p* ≤ 0.001. DHFR, dihydrofolate reductase; DMSO, dimethyl sulfoxide; ns, not significant; PCA, principal component analysis.

Based on these data, and the ability of both folinic acid and thymidine to rescue the STAT3-inhibitory effect of pyrimethamine, we wished to determine whether pyrimethamine caused intracellular metabolite shifts that are consistent with DHFR inhibition in STAT3-driven cancer cells. To do this, we monitored folate metabolism and nucleotide synthesis by metabolite profiling in MDA-MB-468 cells treated with DMSO, methotrexate, or pyrimethamine. As a control, we used ruxolitinib, which blocks STAT3 tyrosine phosphorylation and thus inhibits STAT3-dependent transcription by a completely independent mechanism. Principal component analysis of the metabolic changes following these treatments indicated that pyrimethamine-treated cells clustered with methotrexate-treated cells, and both were different from DMSO-treated and ruxolitinib-treated cells (Fig. 7*A*). This result indicates that pyrimethamine and methotrexate induce similar metabolic perturbation in treated cells and implies a shared target and mechanism of these drugs. Furthermore, we found that among the top 25 differentially changed metabolites within the four treatment groups, nucleotides and nucleotide intermediates were highly represented. In both pyrimethamine and methotrexate clusters, nucleotides were reduced, whereas nucleotide intermediates were increased relative to the DMSO-treated and ruxolitinib-treated samples (Fig. 7*B*). Specifically, deoxyuridine monophosphate significantly accumulated in cells treated with pyrimethamine or methotrexate compared with cells treated with DMSO or ruxolitinib (Fig. 7*C*), corroborating the hypothesis that pyrimethamine, like methotrexate, inhibits the enzyme DHFR. In addition, pyrimethamine and methotrexate treatments resulted in a significant increase in two purine synthesis intermediates that depend on DHFR activity, requiring the incorporation of reduced folate (10-formyl-tetrahydrofolate [THF]), 5-aminoimidazole-4-carboxamide ribonucleotide, and glycineamide ribonucleotide (Fig. 7*C*). Nucleotides related to either *de novo* purine synthesis or the purine salvage pathway such as CDP, GDP, ADP, hypoxanthine, and inosine were also modulated to various extents by pyrimethamine treatment (Fig. 7*C*). Taken together with the folinic acid and thymidine rescue experiments, and the relatively specific effects of pyrimethamine on STAT3-dependent transcription, these findings suggest that the STAT3-inhibitory effects of pyrimethamine are the result of a deficiency in the reduced folate forms, 5,10-methylene-THF and 10-formyl-, downstream of DHFR inhibition, and suggest a unique connection between folate metabolism and STAT3-dependent transcription.

## Discussion

In this study, we have characterized pyrimethamine and methotrexate as unique STAT3 transcriptional inhibitors that function by blocking DHFR activity. Although pyrimethamine has been shown to inhibit DHFR activity in parasites, it was not thought to inhibit human DHFR because of substantial differences in amino acid sequence. However, we have shown that pyrimethamine can stabilize human DHFR to heat denaturation (Fig. 2) inhibit human DHFR activity *in vitro* (Fig. 3) and alter cellular metabolites in a nearly identical manner as methotrexate (Fig. 7), indicating that pyrimethamine is a human DHFR inhibitor. Moreover, our results align with a recently published study that used *in silico* modeling and DHFR activity assays to demonstrate that pyrimethamine plays an anticancer role, in part, by targeting human DHFR (16).

This newly defined role of pyrimethamine as a human DHFR inhibitor led us to test whether DHFR acts as a STAT3 transcriptional coactivator, potentially providing the link between DHFR inhibition and STAT3 inhibition. Although connections between folate and transcriptional regulation have been described, the direct link between DHFR and STAT3 transcriptional activity had not been investigated. Hansen *et al.* (26) have demonstrated that both folic acid and folinic acid can activate STAT3; however, the mechanism appears to be independent of DHFR, relying instead on signaling through folate receptor α (FRα), glycoprotein 130, and JAK to mediate folate-dependent STAT3 phosphorylation. Moreover, the folate metabolism enzyme, methylene-THF dehydrogenase, cyclohydrolase, and formyl-THF synthetase 1, has recently been shown to associate with chromatin and regulate gene expression (27). However, similar studies have yet to be completed for nuclear DHFR (28). Thus, our findings that loss of DHFR *via* siRNA targeting or pyrimethamine treatment dampens STAT3 transcriptional activity without significantly reducing STAT3 tyrosine phosphorylation (Figs. 2 and 3) indicate that DHFR can activate STAT3 transcriptional activity downstream of tyrosine phosphorylation; however, the exact mechanism of activation remains unclear.

Like pyrimethamine, methotrexate has also been described as both a human DHFR and STAT3 inhibitor; however, its role as a STAT3 inhibitor is poorly understood. One piece of evidence supporting methotrexate as a STAT3 inhibitor comes from a prospective phase II study evaluating the effect of immunosuppressive therapy in large granular lymphocytic (LGL) leukemia (29). STAT3 is constitutively active in LGL leukemia, with approximately 40% of LGL leukemia patients having somatic STAT3-activating mutations (30). This prospective study demonstrated that patients with STAT3 mutations were more likely to respond to methotrexate. Moreover, the response scaled with the ability of the STAT3 mutation to convey STAT3 transcriptional activity. These correlative results indicate that methotrexate is a putative STAT3 inhibitor rather than just a broad immunosuppressive agent.

In addition, Thomas *et al.* (31) have shown that methotrexate inhibits JAK/STAT signaling by reducing constitutive but not cytokine-stimulated STAT3 tyrosine phosphorylation in a subset of cell lines. They further demonstrated that this methotrexate-mediated suppression of STAT3 phosphorylation was unaffected by the addition of 0.3 μg/ml of folinic acid. Based on these findings, they concluded that methotrexate inhibits STAT3 phosphorylation, albeit independently of DHFR inhibition. Interestingly, we also saw a methotrexate-dependent decrease in constitutive STAT3 phosphorylation (Fig. 3*B*). However, in our system, methotrexate also inhibited cytokine-stimulated STAT3 activity (Fig. 4, *C* and *D*), and the inhibitory effect was rescued by folinic acid, albeit at much higher concentrations than 0.3 μg/ml (Fig. 5). Moreover, the combination of pyrimethamine and methotrexate had no additive effect on STAT3 inhibition, suggesting that pyrimethamine and methotrexate inhibit STAT3 activity *via* the same molecular target (Fig. 4*D*). Thus, we conclude that methotrexate inhibits STAT3 transcriptional activity *via* DHFR and has a similar mechanism of action to that of pyrimethamine.

That being said, the functional differences between methotrexate and pyrimethamine indicate that the mechanisms of action are not identical. First, methotrexate is an extremely toxic chemotherapeutic agent, whereas pyrimethamine is a generally safe antiparasitic compound. One potential explanation for these differing toxicity profiles is that methotrexate is a much more potent DHFR and STAT3 inhibitor than pyrimethamine (Figs. 2, 3, and 5 and Table 1). This difference in potency may be due to structural differences between the two compounds. Methotrexate is a classical DHFR inhibitor characterized by an intact glutamate side chain that can undergo polyglutamylation by folylpolyglutamate synthase (32). These methotrexate polyglutamates can not only demonstrate prolonged intracellular retention times but also inhibit other folate metabolism enzymes, including TS (33, 34, 35). In contrast, pyrimethamine contains a lipophilic terminal group that cannot be polyglutamylated. Thus, pyrimethamine likely has shorter intracellular retention times and fewer molecular targets (11), leading to a less potent inhibitory effect on folate metabolism in general.

Another key difference between pyrimethamine and methotrexate as STAT3 inhibitors is that methotrexate inhibits STAT3 tyrosine phosphorylation at concentrations required for STAT3 inhibition (Fig. 3*B*). Again, this mechanistic difference may be explained by the structural differences between the two compounds. Classical DHFR inhibitors like methotrexate resemble the structure of endogenous folates and thus are actively taken up *via* folate carriers, transporters, and receptors, including FRα (36, 37). Accordingly, it seems plausible that methotrexate, as a classical DHFR inhibitor, could block FRα-mediated STAT3 phosphorylation, as previously described. In contrast, nonclassical DHFR inhibitors, such as pyrimethamine, do not interact with folate receptors, but rather enter cells *via* passive or facilitated diffusion. Thus, unlike methotrexate, pyrimethamine likely does not interfere with FRα-mediated uptake of endogenous folates and thus is not predicted to reduce folate-dependent STAT3 phosphorylation.

The last apparent mechanistic difference between the two compounds is that the STAT3-inhibitory effects of pyrimethamine, but not methotrexate, can be rescued by thymidine (Fig. 6). Thymidine nucleotide pools are maintained through two distinct cellular pathways. The *de novo* pathway uses 5,10-methylene-THF, a folate species downstream of DHFR, to directly convert deoxyuridine monophosphate to deoxythymidine monophosphate (dTMP) in a reaction catalyzed by TS (38). As DHFR inhibitors, pyrimethamine and methotrexate are presumed to block this *de novo* synthesis pathway by depleting intracellular levels of 5,10-methylene-THF (Fig. 7). In addition, folinic acid is predicted to rescue *de novo* synthesis in the absence of functional DHFR by restoring 5,10-methylene-THF pools in a DHFR-independent manner. Our findings demonstrating the ability of folinic acid to rescue the STAT3-inhibitory effects of both compounds align with these predictions and suggest that DHFR plays a role in regulating STAT3 transcriptional activity.

**Figure 8:**
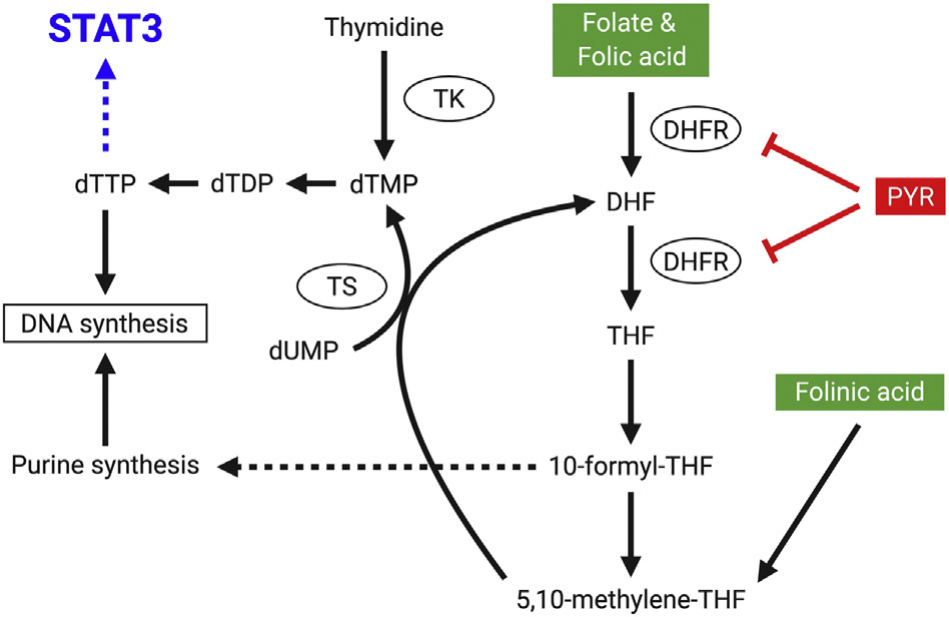
**Mechanistic model of pyrimethamine (PYR) as a DHFR-dependent STAT3 inhibitor.** PYR inhibits DHFR and reduces the intracellular pool of 5,10-methylene-THF needed for the *de novo* synthesis of thymidylate. Insufficient thymidylate levels lead to decreased STAT3 transcriptional activity. PYR-mediated STAT3 inhibition can be rescued by the addition of folinic acid or thymidine, both of which replenish intracellular thymidylate levels. Created with BioRender. DHF, dihydrofolate; DHFR, dihydrofolate reductase; dTDP, deoxythymidine diphosphate; dTMP, deoxythymidine monophosphate; dTTP, deoxythymidine triphosphate; dUMP, deoxyuridine monophosphate; STAT3, signal transducer and activator of transcription 3; THF, tetrahydrofolate; TK, thymidine kinase; TS, thymidylate synthase.

In contrast, the salvage synthesis pathway bypasses DHFR by directly converting cellular thymidine into dTMP *via* the enzymatic activity of thymidine kinase. As the name implies, this salvage pathway allows cells to produce dTMP, which is then converted into deoxythymidine triphosphate and used for DNA synthesis, even in the absence of *de novo* synthesis. Therefore, the ability of both folinic acid and thymidine to rescue the STAT3-inhibitory effects of pyrimethamine implicates DHFR-mediated thymidine biosynthesis, specifically the synthesis of dTMP or some downstream product, in the regulation of STAT3 transcriptional activity (Fig. 8). Furthermore, the unexpected finding that folinic acid, but not thymidine, can rescue the STAT3-inhibitory effects of methotrexate raises the possibility that, unlike pyrimethamine, methotrexate can inhibit both the *de novo* and salvage thymidine synthesis pathways. Interestingly, Abonyi *et al.* (39) have demonstrated that methotrexate can indirectly decrease thymidine kinase activity; however, since the mechanism is thought to rely on DHFR, it will be important to determine whether pyrimethamine has similar effects.

It should be noted that the magnitude of inhibition of STAT3-dependent genes by pyrimethamine is in the range of 30 to 70%. This is not unusual for endogenous genes that are coordinately regulated by a number of basal and inducible transcription factors. However, this is mechanistically relevant, as a drug like TG101 that completely blocks the activating tyrosine phosphorylation of STAT3 leads to a similar magnitude change. Furthermore, it is biologically (and therapeutically) relevant, as loss of STAT3 expression or transcriptional function in these human tumor models abrogates the malignant transformation of these cells.

A key translational question is whether the anticancer effects of pyrimethamine are mediated solely by STAT3 inhibition or whether STAT3-independent effects of DHFR inhibition are playing a significant role. In clinical trials for both its antimicrobial and anticancer effects, pyrimethamine has shown essentially no toxicity at therapeutic levels (10), suggesting that it is not generally cytotoxic at these concentrations. This is in sharp contrast to methotrexate, which can cause a variety of toxicities with continued use. However, since it is not possible to genetically ablate STAT3 from STAT3-dependent cells, it is difficult to discern whether all the anticancer effects of pyrimethamine are STAT3 dependent. As data from clinical trials of pyrimethamine become available, it will be important to determine whether the therapeutic activity of this drug correlates with the magnitude of transcriptional activation of STAT3 in tumor cells.

In this context, the therapeutic relevance of pyrimethamine in cancers characterized by frequent STAT3 activation is becoming more apparent. Pyrimethamine showed on-target activity, as assessed by inhibition of expression of STAT3-dependent genes, in a clinical trial of chronic lymphocytic leukemia (10), a disease in which the leukemia cells are nearly always driven by increased STAT3 transcriptional activity. Thus, understanding the mechanistic properties of pyrimethamine that underlie its effects on STAT3 inhibition may suggest opportunities to further potentiate its therapeutic effects.

In summary, we have identified human DHFR as one of the direct molecular targets responsible for the STAT3-inhibitory effects of pyrimethamine. Moreover, we have demonstrated that folate-mediated thymidine biosynthesis plays a previously unknown role in regulating STAT3 transcriptional activity. Overall, these findings reveal a regulatory node within the STAT3 pathway that may be important for the initiation and treatment of STAT3-driven cancers.

## Experimental procedures

### PISA

PISA was performed by the Chemical Proteomics facility of Karolinska Institute (Department of Medical Biochemistry and Biophysics, Biomedicum, Stockholm), part of SciLifeLab and the Swedish national infrastructure for biological mass spectrometry (MS). PISA, invented and developed at this facility, was performed according to previously published procedures (11). Briefly, the treatment of cells was performed in culture for 2 h in regular medium. After cell detachment, each biological replicate was washed and suspended in PBS supplemented with protease inhibitors (Roche), then split into 15 equal portions for temperature treatment at a range of 43 to 57 °C with 1 °C intervals. The temperature treatment was performed for 3 min at each temperature using a SimplyAmp Thermal Cycler (Applied Biosystems). Samples were then left at room temperature for 6 min before being snap frozen in liquid nitrogen. Cellular lysates were isolated in PBS supplemented with protease inhibitors (Roche) by four cycles of freezing cells in liquid nitrogen and then thawing at 35 °C. For each biological replicate, the 15 equal aliquots were combined in one pool, and sedimentation of unfolded proteins was performed at 150,000*g* for 30 min at 4 °C using an Optima XPN-80 Ultracentrifuge (Beckman Coulter). After measuring the total protein concentration of the soluble fraction by Micro BCA Protein Assay Kit (Thermo Fisher Scientific), equal protein amounts were reduced with dithiothreitol and alkylated with iodoacetamide. Cold acetone protein precipitation was then performed. Protein precipitates were suspended in 8 M urea in 20 mM 4-(2-hydroxyethyl)-1-piperazinepropanesulfonic acid buffer (pH 8.2) and then diluted to 4 M urea. LysC enzyme (Wako) was added and incubated at 30 °C for 8 h. After diluting to 1 M urea, sequencing-grade modified trypsin (Promega) was added, and samples were digested overnight at 37 °C. Each sample was labeled using TMT10 (Thermo Fisher Scientific) for quantitative LC–MS/MS proteomics analysis. One TMT10 hosted two PISA experiments, with four replicates of the vehicle control plus two triplicates for two different drugs (one is not object of this study). Sample desalting and cleaning was performed using Sep-Pak C18 (Waters). The TMT10-labeled peptides were separated using off-line reverse phase UPLC at high pH on a C18 column with a flow rate of 200 μl per minute. Peptides were eluted using a binary gradient of water and acetonitrile, and the eluate was collected into 96 fractions of 100 μl each, concatenated into 12 fractions. Each fraction was analyzed by nano-HPLC–electrospray ionization, and MS/MS analyses were performed using a Q Exactive HF Mass Spectrometer (Thermo Fisher Scientific). Protein identification and quantification was performed using MaxQuant software (Max Planck Institute of Biochemistry) (40) and the UniProt complete human proteome database (UP000005640). A 1% false discovery rate was used as a filter at both protein and peptide levels. After removing contaminants, only proteins with at least two unique peptides were included in the final dataset. Quantification values for each peptide were normalized to the total ion abundance of each TMT reporter and then to the average value for the untreated sample. Two-tailed Student's *t* test (with equal or unequal variance depending on *F* test) was applied to calculate *p* values.

### Cell culture

STAT1-null U3A human fibrosarcoma cells (kindly provided by George Stark; Cleveland Clinic) stably transfected with STAT3-dependent luciferase (41) were maintained in Dulbecco's modified Eagle's medium (DMEM) (Thermo Fisher Scientific) containing 10% fetal bovine serum. MDA-MB-468 human mammary carcinoma cells (kindly provided by Myles Brown; Dana-Farber Cancer Institute) were maintained in DMEM (Thermo Fisher Scientific) containing 10% fetal bovine serum. SKBR3 human mammary carcinoma cells (kindly provided by Lyndsay Harris; Dana-Farber Cancer Institute) were maintained in RPMI (Thermo Fisher Scientific) with 10% fetal bovine serum. All cells were maintained in a humidified incubator at 37 °C with 5% CO_2_.

### Compounds

Pyrimethamine (Sigma–Aldrich) and methotrexate (3507; Santa Cruz Biotechnology) were prepared at 25 mM in DMSO. TG101 (364740; Santa Cruz Biotechnology) was prepared at 10 mM in DMSO. Cells were treated as indicated.

### CETSA

U3A cells were cultured in DMEM (Thermo Fisher Scientific) with 10% heat-inactivated fetal bovine serum and 1% penicillin/streptomycin. Intact U3A cells were treated with pyrimethamine at a concentration of 10 μM or DMSO (0.1% v/v final) for 10 min at 37 °C. Cells were washed once with PBS, harvested using trypsin, and centrifuged at 300*g*. The pellet was resuspended in Tris-buffered saline (TBS) supplemented with cOmplete protease inhibitor cocktail (Roche), aliquoted (50 μl; 8 × 10^5^ cells per temperature) into PCR strip tubes, and heated at the indicated temperature in a Veriti Thermal Cycler (Applied Biosystems) for 3 min, then cooled for another 3 min at room temperature. Cells were then lysed by freeze–thawing three times with 3-min incubations in an ethanol/dry ice bath and a water bath at 37 °C. The lysates were then centrifuged at 17,000*g* for 20 min at 4 °C to remove cellular debris and pelleted protein aggregates. Supernatants (40 μl) were transferred to new tubes and prepared for immunoblotting. A similar protocol was used for the treatment of U3A cell lysate. Briefly, U3A cells in exponential growth rate were collected, resuspended in TBS supplemented with cOmplete protease inhibitor cocktail (Roche), lysed by freeze–thaw cycle, and centrifuged at 17,000*g* for 20 min at 4 °C. Supernatant was aliquoted into two tubes and treated with 10 μM pyrimethamine or DMSO for 10 min at room temperature. Treated cell lysates were aliquoted (50 μl) into PCR strip tubes and heated at the indicated temperature in a Veriti Thermal Cycler (Applied Biosystems) for 3 min and then cooled for another 3 min at room temperature. Lysates were then centrifuged at 17,000*g* for 20 min at 4 °C, and 40 μl was transferred to new tubes and prepared for immunoblotting. DHFR stabilization was evaluated with superoxide dismutase 1 as a thermostable loading control. Band intensities were quantified using Fiji (42) and then plotted and fitted in GraphPad Prism (GraphPad Software, Inc) to obtain apparent *T*_agg_ values.

### Immunoblotting

Cells were lysed on ice for 15 min in either radioimmunoprecipitation assay lysis buffer (Boston BioProducts) or EBC lysis buffer (50 mM Tris [pH 8.0], 250 mM NaCl, and 0.5% Nonidet-P40 [NP-40]) containing phosphatase and complete protease inhibitors (Roche). Lysates were cleared *via* centrifugation and quantified using the Bradford Reagent (Bio-Rad Laboratories). Lysates were mixed with 2× sample buffer (0.125 M Tris [pH 6.8], 4% SDS, and 20% glycerol) containing 4% β-mercaptoethanol and boiled for 5 min. Nuclear cytoplasmic fractionations were performed using the Nuclear Extract Kit (Active Motif) according to the manufacturer's protocol. Samples were resolved on 10 to 12% acrylamide gels and transferred to 0.45 μm Amersham Protran nitrocellulose (GE Healthcare). Membranes were blocked with 5% milk in TBS/Tween (Boston BioProducts) and probed with antibodies to pSTAT3 (catalog no.: 9131; Cell Signaling), STAT3 (catalog no.: 482, Santa Cruz Biotechnology; catalog no.: 9139, Cell Signaling), DHFR (catalog no.: 377091; Santa Cruz Biotechnology), tubulin (catalog no.: T5168; Sigma–Aldrich), and superoxide dismutase 1 (catalog no.: 11407; Santa Cruz Biotechnology). Immunoblots were developed, and band intensity was quantified using Fiji (42).

### Apoptosis assay

Cells (5 × 10^5^) were plated in 6-well plates. The next day, cells were treated as indicated. Apoptosis was detected using the FITC Annexin V Apoptosis Detection Kit I (BD Biosciences) and quantified using an LSRFortessa (BD Biosciences).

### Viability assay

Cells (3–5 × 10^3^) were plated in white opaque 96-well plates. The next day, cells were treated as indicated. ATP levels were detected at the indicated time points as a proxy for number of viable cells using the CellTiter-Glo Luminescent Cell Viability Assay (Promega). Luminescence was measured using a Luminoskan Ascent Microplate Reader (Labsystems).

### RNA interference

Cells were transfected with 10 nM of siRNA targeting CHCHD2 (catalog no.: 89755; Santa Cruz Biotechnology), DHFR (catalog no.: 37078; Santa Cruz Biotechnology), STAT3 (catalog no.: D-003544-03-0010; Dharmacon), SCL/TAL1 interrupting locus (catalog no.: 4775; Santa Cruz Biotechnology), GTPase-activating Rap/Ran-GAP domain–like 1 (catalog no.: 92345; Santa Cruz Biotechnology), or nontargeting siRNAs designated as siConA (catalog no.: 37007; Santa Cruz Biotechnology), and siCon2 (catalog no.: D-001210-02-05; Dharmacon) using Lipofectamine RNAiMax (Invitrogen). Cells were transfected for the indicated amounts of time prior to beginning biological experiments.

### Cytokine stimulation

Cells were stimulated with 10 ng/ml interleukin 6 (Peprotech) or 10 ng/ml OSM (Peprotech). Cells were stimulated for 15 min for whole cell lysate immunoblotting, 60 to 90 min for qRT–PCR, and 5 to 6 h for luciferase reporter assays.

### Luciferase reporter assay

U3A cells (3 × 10^3^) were plated in white opaque 96-well plates and allowed to adhere overnight. For siRNA-mediated knockdown, cells were left in the incubator for 48 h. For drug-mediated inhibition, cells were pretreated with compound for 1 h. Cells were then stimulated with cytokine for 5 to 6 h to activate STAT3. Luciferase activity was detected using the Bright-Glo Luciferase Assay System (Promega) and quantitated using a Luminoskan Ascent Microplate Reader (Labsystems).

### qRT–PCR

Total RNA was extracted from cells using the RNeasy Mini Kit (Qiagen). Complementary DNA was generated using the TaqMan Reverse Transcription Reagents (Invitrogen). qPCR was performed in triplicate or quadruplicate using the Power SYBR Green PCR Master Mix (Invitrogen) on a 7500 Real-Time PCR System (Applied Biosystems) or a QuantStudio 6 Flex Real-Time PCR System (Applied Biosystems). Specificity of amplification was confirmed by melting curve analysis. Cycle threshold (C_T_) values for target genes were normalized to the endogenous reference gene GAPDH. Primer sequences (Table S3) were designed from the University of California Santa Cruz genome browser reference mRNA sequences using Primer3 (43).

### ITC

ITC was performed on a MicroCal Auto-iTC200 calorimeter (Malvern Panalytical). Experiments were performed at 25 ^o^C in 20 mM Hepes (pH 7.5), 50 mM NaCl, 0.5% glycerol, and 80 μM Tris(2-carboxyethyl)phosphine. The cell contained 11 μM DHFR in 280 μl volume, and the syringe contained 130 μM pyrimethamine or methotrexate. Both the cell and syringe contained 200 μM NADPH and 1.5% DMSO as additives. Each titration experiment consisted of 18 2 μl injections of pyrimethamine or methotrexate from the syringe into DHFR in the cell every 180 s with constant stirring at 1000 rpm. As a control, pyrimethamine was also titrated into buffer (with supplemental 200 μM NADPH and 1.5% DMSO) using the same injection parameters to determine the heat of dilution. Raw data were analyzed with the corresponding Origin 7 software (OriginLab), and the background-subtracted isotherm was fit to the one-site model (44).

### DHFR enzymatic assay

DHFR enzymatic activity assay was performed for methotrexate and pyrimethamine by monitoring NADPH absorbance at 340 nm. Briefly, flat bottomed 96-well clear plates were prepared to a final volume of 200 μl with inhibitors (pyrimethamine: 0.97–2000 μM and methotrexate: 0.0012–2.5 μM), DHF (137.5 μM), DHFR (200 nM), and NADPH (125 μM). The NADPH was added last, and immediately following the addition, the plate was loaded into a BioTek Synergy Mx Microplate Reader, where it was incubated at 37 °C. Absorbance readings were made every 5 min for up to 180 min. IC_50_ values were calculated using the 30 min time point. Percent inhibition values were calculated using the following equation:

$$\text{\%}\;\text{Inhibition}=\frac{\text{A}340\;\text{Inhibitor}-\text{A}340\;\text{“Vehicle”}}{\text{A}340\;\text{“No}\;\text{DHFR”}-\text{A}340\;\text{“Vehicle”}}\times 100\text{\%}$$

Where “Vehicle” refers to wells loaded with DMSO (5%), DHFR (200 nM), DHF (137.5 μM), and NADPH (125 μM), and the “No DHFR” consisted of wells containing DMSO (5%), DHF (137.5 μM), and NADPH (125 μM). Percent inhibition was plotted *versus* concentration of inhibitor and fit using GraphPad Prism, version 9.0. Experiments were performed using duplicate or triplicate wells, and three independent experiments were performed (n = 3). IC_50_ values are reported as the mean ± standard deviation between experiments. Notably, pyrimethamine experiences solubility issues at concentrations above 1 mM in 5% DMSO, which likely explains the slight drop in inhibition at the highest concentrations. Solubility issues also contribute to the variability between experiments.

### Chromatin IP

Cells (1.5 × 10^7^) were fixed in 1% formaldehyde for 10 min at room temperature, followed by quenching of the formaldehyde with 0.125 M glycine. Cells were washed with PBS (Invitrogen), and nuclei were isolated in 400 μl of cell lysis buffer (10 mM Tris [pH 8.0], 10 mM NaCl, and 0.2% NP-40) containing phosphatase and complete protease inhibitors (Roche). Nuclei were centrifuged at 660*g* for 5 min at 4 °C and lysed in 300 μl of nuclear lysis buffer (50 mM Tris [pH 8.1], 10 mM EDTA, 1% SDS, and phosphatase and protease inhibitors). Chromatin was sheared to an average size of 500 to 1000 base pairs using a Fisher Scientific Sonic Dismembranator Model 500 PDQ on setting 15 in 15 s pulses. Debris was removed by centrifugation at 20,800*g* for 10 min at 4 °C, 700 μl of IP dilution buffer (20 mM Tris [pH 8.1], 2 mM EDTA, 150 mM NaCl, 1% Triton X-100, and 0.01% SDS) was added, and 150 to 200 μl of lysate was immunoprecipitated overnight at 4 °C with 1 μg of antibody to STAT3 (catalog no.: 482; Santa Cruz Biotechnology) or RNA pol II (catalog no.: 9001; Santa Cruz Biotechnology). Lysate was then added to 60 μl of Protein A/G PLUS agarose beads (catalog no.: 2003; Santa Cruz Biotechnology) and rotated for 4 to 6 h at 4 °C. Beads were washed three times with IP wash I (20 mM Tris [pH 8.1], 2 mM EDTA, 50 mM NaCl, 1% Triton X-100, 0.1% SDS), once with IP wash II (10 mM Tris [pH 8.1], 1 mM EDTA, 250 mM LiCl, 1% NP-40, and 1% sodium deoxycholate), and three times with TE buffer (G-Biosciences). Beads were resuspended in 150 μl elution buffer (100 mM sodium bicarbonate and 1% SDS) and incubated overnight at 65 °C. DNA was purified using a PCR Purification Kit (Qiagen) according to the manufacturer's protocol. qPCR was performed using the indicated primers (Table S3). Results were normalized to input.

### LC/MS-based metabolite profiling

#### Sample preparation for LC/MS analysis of polar metabolites from MDA-MB-468 cells

For characterization by MS, MDA-MB-468 cells were treated with DMSO, 0.05 μM methotrexate, 10 μM pyrimethamine, and 1 μM ruxolitinib for 24 h, respectively. Per condition, technical triplicates of one million cells were harvested, washed briefly in 0.9% NaCl (high-grade salt and LC/MS-grade water [catalog no.: W6500, Fisher Scientific or catalog no.: 1.15333, Sigma–Aldrich]), and extracted in 500 μl prechilled extraction buffer (80% LC/MS-grade methanol, 20% 125 mM ammonium acetate, 12.5 mM sodium ascorbate prepared in LC/MS-grade water, and supplemented with aminopterin (catalog no.: 16.330; Schircks Laboratories) and isotopically labeled internal standards (17 amino acids and reduced glutathione [Cambridge Isotope Laboratories; MSK-A2-1.2 and CNLM-6245-10])). After centrifugation for 10 min, 4 °C, at maximum speed on a benchtop centrifuge (Eppendorf), the cleared supernatant was transferred to a new tube and dried using a nitrogen dryer (Reacti-Vap Evaporator; Thermo Fisher Scientific; catalog no.: TS-18826) while on ice. Once the drying process was completed, samples were reconstituted in 50 μl QReSS water (Cambridge Isotope Laboratories; catalog no.: MSK-QRESS-KIT) by brief sonication in a 4 °C water bath. Extracted metabolites were spun for 3 min, 4 °C, at maximum speed on a bench-top centrifuge, and the cleared supernatant was transferred to LC/MS microvials (National Scientific; catalog no.: C5000-45B). A small amount of each sample was pooled and serially diluted 3-fold and 10-fold to be used as quality controls throughout the run of each batch.

#### Chromatographic conditions for LC/MS

One microliter (equivalent to 20,000 cells) of reconstituted sample was injected into a ZIC-pHILIC 150 × 2.1 mm (particle size of 5 μm) column (EMD Millipore) operated on a Vanquish Flex UHPLC Systems (Thermo Fisher Scientific). Chromatographic separation was achieved using the following conditions: buffer A was acetonitrile; buffer B was 20 mM ammonium carbonate, and 0.1% ammonium hydroxide. Gradient conditions were linear gradient from 20% to 80% B; 20 to 20.5 min: from 80% to 20% B; 20.5 to 28 min: hold at 20% B. The column oven and autosampler tray were held at 25 and 4 °C, respectively.

#### Orbitrap conditions for targeted analysis of polar metabolites

MS data acquisition was performed using a Q Exactive benchtop orbitrap mass spectrometer equipped with an Ion Max source and a HESI II probe (Thermo Fisher Scientific) and was performed in positive and negative ionization mode in a range of *m/z* = 70 to 1000, with the resolution set at 70,000, the automatic gain control target at 1 × 10^6^, and the maximum injection time at 20 ms. For nucleotide target selective ion monitoring scans, the resolution was set at 70,000, the automatic gain control target was 1 × 10^5^, and the maximum injection time was 100 ms.

Relative quantitation of polar metabolites was performed with TraceFinder 4.1 (Thermo Fisher Scientific) using a 5 ppm mass tolerance and referencing an in-house library of chemical standards (submitted separately). Pooled samples and fractional dilutions were prepared as quality controls, and only those metabolites were taken for further analysis, for which the correlation between the dilution factor and the peak area was >0.95 (high confidence metabolites) and for which the coefficient of variation was below 30%. Normalization for biological material amounts was based on the total integrated peak area values of high-confidence metabolites within an experimental batch after normalizing to the averaged factor from all mean-centered chromatographic peak areas of isotopically labeled amino acid internal standards. The data were log transformed and Pareto scaled within the MetaboAnalyst-based statistical analysis platform (45). Both principal component analysis and heatmap analysis were performed using the MetaboAnalyst online platform. Individual *t* tests were performed in Prism software.

### Computational analyses

The *in vitro* sensitivity data for pyrimethamine and methotrexate were obtained from the Sanger drug sensitivity IC_50_ dataset available through the Cancer Dependency Map Data Explorer (Broad Institute) (17) and plotted using the Plotly Chart Studio. Pearson correlation coefficients were computed between pyrimethamine (Genomics of Drug Sensitivity in Cancer: 71) and each of the compounds available in the Sanger dataset. The top two correlates are presented.

### Statistical analyses

Statistical tests indicated in the figure legends were performed using GraphPad Prism 8.1.2 and 8.2.0. Values of *p* ≤ 0.05 were considered significant (not significant [ns]; *p* > 0.05, ∗*p* ≤ 0.05, ∗∗*p* ≤ 0.01, and ∗∗∗*p* ≤ 0.001). Data are presented as mean ± SD for one representative experiment. Most experiments were completed at least twice.

## Data availability

PISA or metabolomic data not included in the article will be shared upon request. Please contact the corresponding author.

## Supporting information

This article contains supporting information.

## Conflict of interest

The authors declare that they have no conflicts of interest with the contents of this article.

## References

[1] Miklossy G., Hilliard T.S., Turkson J. (2013). Therapeutic modulators of STAT signalling for human diseases. Nat. Rev. Drug Discov..

[2] Fagard R., Metelev V., Souissi I., Baran-Marszak F. (2013). STAT3 inhibitors for cancer therapy: Have all roads been explored?. JAKSTAT.

[3] Furqan M., Akinleye A., Mukhi N., Mittal V., Chen Y., Liu D. (2013). STAT inhibitors for cancer therapy. J. Hematol. Oncol..

[4] Furtek S.L., Backos D.S., Matheson C.J., Reigan P. (2016). Strategies and approaches of targeting STAT3 for cancer treatment. ACS Chem. Biol..

[5] Wong A.L.A., Hirpara J.L., Pervaiz S., Eu J.Q., Sethi G., Goh B.C. (2017). Do STAT3 inhibitors have potential in the future for cancer therapy?. Expert Opin. Investig. Drugs.

[6] Qin J.J., Yan L., Zhang J., Zhang W.D. (2019). STAT3 as a potential therapeutic target in triple negative breast cancer: A systematic review. J. Exp. Clin. Cancer Res..

[7] Bai L., Zhou H., Xu R., Zhao Y., Chinnaswamy K., McEachern D., Chen J., Yang C.Y., Liu Z., Wang M., Liu L., Jiang H., Wen B., Kumar P., Meagher J.L. (2019). A potent and selective small-molecule degrader of STAT3 achieves complete tumor regression in vivo. Cancer Cell.

[8] Takakura A., Nelson E.A., Haque N., Humphreys B.D., Zandi-Nejad K., Frank D.A., Zhou J. (2011). Pyrimethamine inhibits adult polycystic kidney disease by modulating STAT signaling pathways. Hum. Mol. Genet..

[9] Nelson E.A., Sharma S.V., Settleman J., Frank D.A. (2011). A chemical biology approach to developing STAT inhibitors: Molecular strategies for accelerating clinical translation. Oncotarget.

[10] Brown J.R., Walker S.R., Heppler L.N., Tyekucheva S., Nelson E.A., Klitgaard J., Nicolais M., Kroll Y., Xiang M., Yeh J.E., Chaudhury M., Giaccone Z.T., Fernandes S.M., Jacobsen E.D., Fisher D.C. (2021). Targeting constitutively active STAT3 in chronic lymphocytic leukemia: A clinical trial of the STAT3 inhibitor pyrimethamine with pharmacodynamic analyses. Am. J. Hematol..

[11] Gaetani M., Sabatier P., Saei A.A., Beusch C.M., Yang Z., Lundström S.L., Zubarev R.A. (2019). Proteome integral solubility alteration: A high-throughput proteomics assay for target deconvolution. J. Proteome Res..

[12] Savitski M.M., Reinhard F.B., Franken H., Werner T., Savitski M.F., Eberhard D., Martinez Molina D., Jafari R., Dovega R.B., Klaeger S., Kuster B., Nordlund P., Bantscheff M., Drewes G. (2014). Tracking cancer drugs in living cells by thermal profiling of the proteome. Science.

[13] Almqvist H., Axelsson H., Jafari R., Dan C., Mateus A., Haraldsson M., Larsson A., Martinez Molina D., Artursson P., Lundbäck T., Nordlund P. (2016). CETSA screening identifies known and novel thymidylate synthase inhibitors and slow intracellular activation of 5-fluorouracil. Nat. Commun..

[14] Zhang K., Rathod P.K. (2002). Divergent regulation of dihydrofolate reductase between malaria parasite and human host. Science.

[15] Nzila A., Ward S.A., Marsh K., Sims P.F., Hyde J.E. (2005). Comparative folate metabolism in humans and malaria parasites (part II): Activities as yet untargeted or specific to Plasmodium. Trends Parasitol..

[16] Liu H., Qin Y., Zhai D., Zhang Q., Gu J., Tang Y., Yang J., Li K., Yang L., Chen S., Zhong W., Meng J., Liu Y., Sun T., Yang C. (2019). Antimalarial drug pyrimethamine plays a dual role in antitumor proliferation and metastasis through targeting DHFR and TP. Mol. Cancer Ther..

[17] Tsherniak A., Vazquez F., Montgomery P.G., Weir B.A., Kryukov G., Cowley G.S., Gill S., Harrington W.F., Pantel S., Krill-Burger J.M., Meyers R.M., Ali L., Goodale A., Lee Y., Jiang G. (2017). Defining a cancer dependency map. Cell.

[18] Chu E., Takimoto C.H., Voeller D., Grem J.L., Allegra C.J. (1993). Specific binding of human dihydrofolate reductase protein to dihydrofolate reductase messenger RNA in vitro. Biochemistry.

[19] Ercikan-Abali E.A., Banerjee D., Waltham M.C., Skacel N., Scotto K.W., Bertino J.R. (1997). Dihydrofolate reductase protein inhibits its own translation by binding to dihydrofolate reductase mRNA sequences within the coding region. Biochemistry.

[20] Ducker G.S, Rabinowitz J.D. (2017). One-carbon metabolism in health and disease. Cell Metab..

[21] Shuvalov O., Petukhov A., Daks A., Fedorova O., Vasileva E., Barlev N.A. (2017). One-carbon metabolism and nucleotide biosynthesis as attractive targets for anticancer therapy. Oncotarget.

[22] Grem J.L., Voeller D.M., Geoffroy F., Horak E., Johnston P.G., Allegra C.J. (1994). Determinants of trimetrexate lethality in human colon cancer cells. Br. J. Cancer.

[23] Kaminskas E. (1982). Effects of methotrexate on ribonucleotide pools in growing and in growth-arrested tumor cells and antagonism by RNA synthesis inhibitors. J. Biol. Chem..

[24] Alvarez J.V., Febbo P.G., Ramaswamy S., Loda M., Richardson A., Frank D.A. (2005). Identification of a genetic signature of activated signal transducer and activator of transcription 3 in human tumors. Cancer Res..

[25] Rushworth D., Mathews A., Alpert A., Cooper L.J. (2015). Dihydrofolate reductase and thymidylate synthase transgenes resistant to methotrexate interact to permit novel transgene regulation. J. Biol. Chem..

[26] Hansen M.F., Greibe E., Skovbjerg S., Rohde S., Kristensen A.C., Jensen T.R., Stentoft C., Kjær K.H., Kronborg C.S., Martensen P.M. (2015). Folic acid mediates activation of the pro-oncogene STAT3 via the Folate Receptor alpha. Cell. Signal..

[27] Sdelci S., Rendeiro A.F., Rathert P., You W., Lin J.-M.G., Ringler A., Hofstätter G., Moll H.P., Gürtl B., Farlik M., Schick S., Klepsch F., Oldach M., Buphamalai P., Schischlik F. (2019). MTHFD1 is a genetic interactor of BRD4 and links folate metabolism to transcriptional regulation. Nat. Genet..

[28] Yuan T.T., Huang Y., Zhou C.X., Yu Y., Wang L.S., Zhuang H.Y., Chen G.Q. (2009). Nuclear translocation of dihydrofolate reductase is not a pre-requisite for DNA damage induced apoptosis. Apoptosis.

[29] Loughran T.P., Zickl L., Olson T.L., Wang V., Zhang D., Rajala H.L., Hasanali Z., Bennett J.M., Lazarus H.M., Litzow M.R., Evens A.M., Mustjoki S., Tallman M.S. (2015). Immunosuppressive therapy of LGL leukemia: Prospective multicenter phase II study by the Eastern Cooperative Oncology Group (E5998). Leukemia.

[30] Koskela H.L., Eldfors S., Ellonen P., van Adrichem A.J., Kuusanmäki H., Andersson E.I., Lagström S., Clemente M.J., Olson T., Jalkanen S.E., Majumder M.M., Almusa H., Edgren H., Lepistö M., Mattila P. (2012). Somatic STAT3 mutations in large granular lymphocytic leukemia. N. Engl. J. Med..

[31] Thomas S., Fisher K.H., Snowden J.A., Danson S.J., Brown S., Zeidler M.P. (2015). Methotrexate is a JAK/STAT pathway inhibitor. PLoS One.

[32] Gao T., Zhang C., Shi X., Guo R., Zhang K., Gu J., Li L., Li S., Zheng Q., Cui M., Cui M., Gao X., Liu Y., Wang L. (2019). Targeting dihydrofolate reductase: Design, synthesis and biological evaluation of novel 6-substituted pyrrolo[2,3-d]pyrimidines as nonclassical antifolates and as potential antitumor agents. Eur. J. Med. Chem..

[33] Allegra C.J., Chabner B.A., Drake J.C., Lutz R., Rodbard D., Jolivet J. (1985). Enhanced inhibition of thymidylate synthase by methotrexate polyglutamates. J. Biol. Chem..

[34] Chu E., Drake J.C., Boarman D., Baram J., Allegra C.J. (1990). Mechanism of thymidylate synthase inhibition by methotrexate in human neoplastic cell lines and normal human myeloid progenitor cells. J. Biol. Chem..

[35] Municio C., Soler Palacios B., Estrada-Capetillo L., Benguria A., Dopazo A., García-Lorenzo E., Fernández-Arroyo S., Joven J., Miranda-Carús M.E., González-Álvaro I., Puig-Kröger A. (2016). Methotrexate selectively targets human proinflammatory macrophages through a thymidylate synthase/p53 axis. Ann. Rheum. Dis..

[36] Spinella M.J., Brigle K.E., Sierra E.E., Goldman I.D. (1995). Distinguishing between folate receptor-alpha-mediated transport and reduced folate carrier-mediated transport in L1210 leukemia cells. J. Biol. Chem..

[37] Nogueira E., Sárria M.P., Azoia N.G., Antunes E., Loureiro A., Guimarães D., Noro J., Rollett A., Guebitz G., Cavaco-Paulo A. (2018). Internalization of methotrexate conjugates by folate receptor-α. Biochemistry.

[38] Anderson D.D., Quintero C.M., Stover P.J. (2011). Identification of a de novo thymidylate biosynthesis pathway in mammalian mitochondria. Proc. Natl. Acad. Sci. U. S. A..

[39] Abonyi M., Prajda N., Hata Y., Nakamura H., Weber G. (1992). Methotrexate decreases thymidine kinase activity. Biochem. Biophys. Res. Commun..

[40] Cox J., Mann M. (2008). MaxQuant enables high peptide identification rates, individualized p.p.b.-range mass accuracies and proteome-wide protein quantification. Nat. Biotechnol..

[41] Lynch R.A., Etchin J., Battle T.E., Frank D.A. (2007). A small-molecule enhancer of signal transducer and activator of transcription 1 transcriptional activity accentuates the antiproliferative effects of IFN-gamma in human cancer cells. Cancer Res..

[42] Schindelin J., Arganda-Carreras I., Frise E., Kaynig V., Longair M., Pietzsch T., Preibisch S., Rueden C., Saalfeld S., Schmid B., Tinevez J.Y., White D.J., Hartenstein V., Eliceiri K., Tomancak P. (2012). Fiji: An open-source platform for biological-image analysis. Nat. Methods.

[43] Untergasser A., Cutcutache I., Koressaar T., Ye J., Faircloth B.C., Remm M., Rozen S.G. (2012). Primer3-new capabilities and interfaces. Nucleic Acids Res..

[44] Appleman J.R., Prendergast N., Delcamp T.J., Freisheim J.H., Blakley R.L. (1988). Kinetics of the formation and isomerization of methotrexate complexes of recombinant human dihydrofolate reductase. J. Biol. Chem..

[45] Xia J., Sinelnikov I.V., Han B., Wishart D.S. (2015). MetaboAnalyst 3.0—making metabolomics more meaningful. Nucleic Acids Res..

