# Supplementary material for: The antimicrobial drug pyrimethamine inhibits STAT3 transcriptional activity by targeting the enzyme dihydrofolate reductase
Source: J Biol Chem. 2021 Dec 23;298(2):101531. doi: 10.1016/j.jbc.2021.101531 (PMC8800111; doi:10.1016/j.jbc.2021.101531)
Supplement: Supplemental Figures S1–S3 and Tables S1–S3 [file mmc1.docx]

**The antimicrobial drug pyrimethamine inhibits STAT3 transcriptional activity**

**by targeting the enzyme dihydrofolate reductase**

**Lisa N. Heppler, Sanaz Attarha, Rosanne Persaud, Jennifer I. Brown, Peng Wang,**

**Boryana Petrova, Isidora Tošić, Foster B. Burton, Yael Flamand, Sarah R. Walker,**

**Jennifer E. Yeh, Roman A. Zubarev, Massimiliano Gaetani, Naama Kanarek, Brent D. G. Page, and David A. Frank**

*Supporting Information*—This document includes Figures S1-S3 and Tables S1–S3.

**
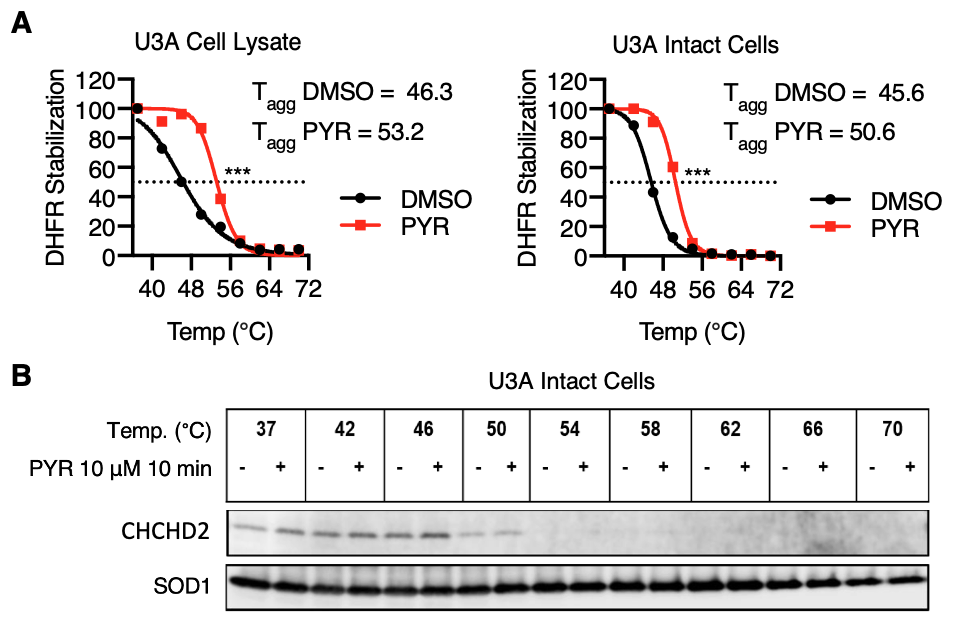
**

**Figure S1.** Pyrimethamine stabilizes DHFR but not CHCHD2 to heat denaturation. (A) DHFR protein levels in Fig 2C were quantified and plotted. DHFR Stabilization presented as % of 37 °C levels. Statistical comparisons were performed between T_agg_ values. (B) U3A intact cells were treated with 10 μM PYR for ten minutes. Following treatment, samples were heated to the indicated temperatures. Soluble protein levels were analyzed by immunoblotting with the indicated antibodies. SOD1 was used as a thermostable control. The CHCHD2 and SOD1 data shown here are from the same samples and immunoblot as shown in Fig 2C, allowing for comparison between DHFR, CHCHD2, and SOD1. ns P > 0.05, * P ≤ 0.05, ** P ≤ 0.01 and *** P ≤ 0.001 with least squares regression with extra sum-of-squares F-test used in (A).

**
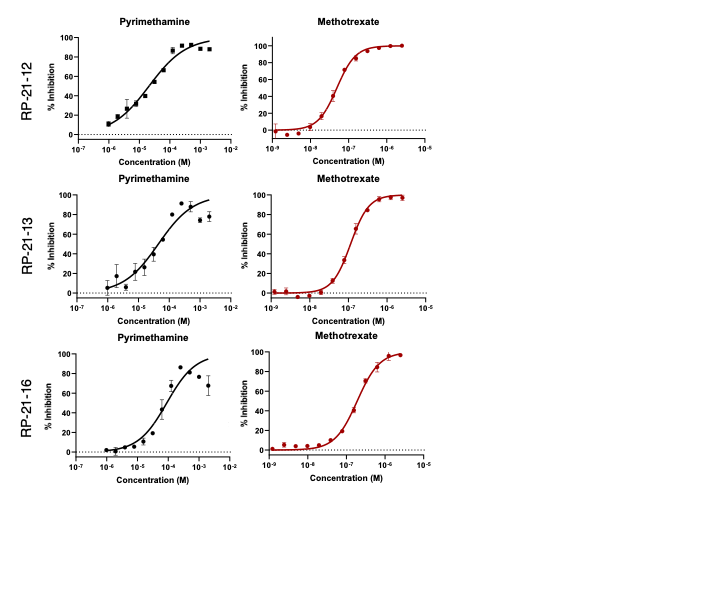
**

**Figure S2.** Pyrimethamine inhibits *in vitro* DHFR enzymatic activity. DHFR enzymatic activity assay was performed for PYR and MTX by monitoring NADPH absorbance at 340 nm. Percent inhibition was plotted versus concentration of inhibitor and curves were fit using Graphpad Prism Version 9.0. Experiments were performed using duplicate or triplicate wells and 3 independent experiments were performed (n=3). See Table 1 for IC_50_ values.

**
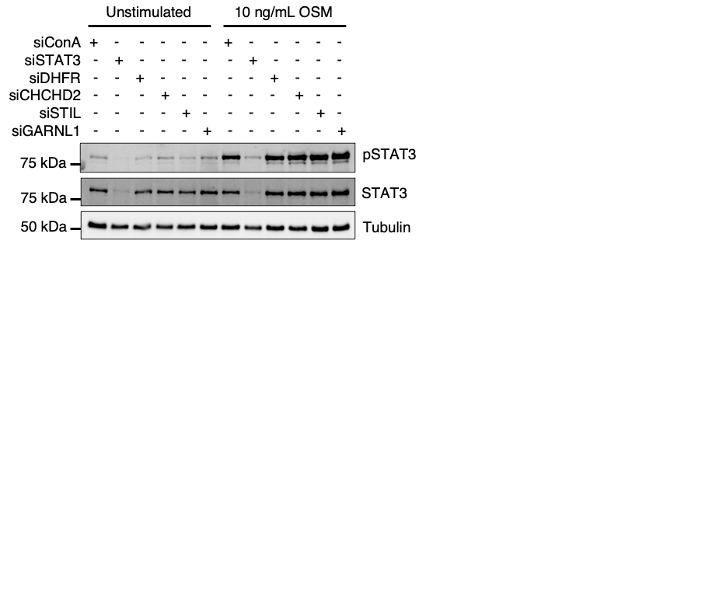
**

**Figure S3. DHFR knockdown has minimal effect on basal and induced STAT3 phosphorylation.** U3A cells were transfected with siRNA targeting DHFR and three other candidates from the PISA screen, specifically CHCHD2, STIL, and GARNL1. siRNA targeting STAT3 was used as a positive control. 48 hours after transfection, cells were stimulated with 10 ng/mL oncostatin M (OSM) for 15 minutes and analyzed by immunoblotting with the indicated antibodies.

**Table S1.** Top 500 proteins stabilized by pyrimethamine in U3A human fibrosarcoma cells.

| *Gene(s)* | *Unique*  *Peptides* | *Sequence*  *Coverage (%)* | *Sequence*  *Length* | *Log2*  *(PYR/DMSO)* | *-Log10*  *(P-Value)* |
| --- | --- | --- | --- | --- | --- |
| DHFR | 11 | 68.4 | 187 | 0.80 | 2.21 |
| TMEM115 | 2 | 7.7 | 351 | 0.38 | 1.12 |
| ATP1A1 | 7 | 9 | 992 | 0.37 | 0.95 |
| CHCHD2; CHCHD2P9 | 5 | 38.4 | 151 | 0.36 | 2.12 |
| HSPD1 | 43 | 80.6 | 573 | 0.35 | 0.88 |
| STIL | 2 | 2.6 | 1241 | 0.35 | 0.91 |
| CASP8 | 3 | 12.2 | 395 | 0.34 | 0.90 |
| SIAE | 3 | 5.3 | 488 | 0.34 | 0.73 |
| ESYT2 | 2 | 3.6 | 884 | 0.33 | 0.95 |
| H2AFY | 6 | 19.1 | 371 | 0.33 | 1.05 |
| PLCH1 | 2 | 1.1 | 1655 | 0.32 | 0.71 |
| SLC25A5; SLC25A4; SLC25A6; SLC25A31 | 2 | 7 | 298 | 0.31 | 0.79 |
| SMC5 | 2 | 2.1 | 1101 | 0.30 | 0.76 |
| MRPL37 | 5 | 13.5 | 483 | 0.29 | 0.69 |
| ATP2A2; ATP2A1; ATP2A3 | 2 | 3.3 | 997 | 0.28 | 0.64 |
| C11orf73 | 4 | 23.9 | 197 | 0.28 | 0.73 |
| HIST1H4A | 7 | 42.7 | 103 | 0.28 | 0.68 |
| FRYL | 2 | 0.9 | 3013 | 0.28 | 1.10 |
| YKT6 | 6 | 26.8 | 198 | 0.27 | 0.51 |
| HNRNPC | 16 | 53.1 | 290 | 0.27 | 0.70 |
| RFC4 | 12 | 36.9 | 363 | 0.27 | 0.77 |
| LYRM2 | 4 | 42 | 88 | 0.27 | 0.82 |
| MLKL | 9 | 17.8 | 471 | 0.27 | 0.49 |
| LENG8 | 2 | 4.2 | 800 | 0.27 | 0.72 |
| ATP1B3 | 5 | 16.8 | 279 | 0.27 | 0.79 |
| ASMTL | 5 | 10.8 | 563 | 0.27 | 0.59 |
| MYBBP1A | 2 | 1.8 | 1252 | 0.26 | 0.52 |
| ZNF148 | 5 | 9.7 | 794 | 0.26 | 0.66 |
| KIAA2012; SPEG | 2 | 1.4 | 1181 | 0.26 | 0.57 |
| STC1 | 2 | 10.1 | 247 | 0.25 | 1.02 |
| UBE2Z | 3 | 14.6 | 246 | 0.25 | 0.51 |
| MCEE | 3 | 16.5 | 176 | 0.25 | 0.60 |
| HMOX2 | 3 | 14.1 | 370 | 0.25 | 1.14 |
| DDX54 | 3 | 4.3 | 881 | 0.25 | 0.49 |
| MPZL1 | 2 | 13.8 | 145 | 0.25 | 0.60 |
| MRPS2 | 2 | 6.3 | 270 | 0.25 | 0.59 |
| UBE2S | 10 | 66.2 | 222 | 0.25 | 0.67 |
| GLS | 19 | 37.6 | 598 | 0.24 | 0.43 |
| MRPS16 | 2 | 22.6 | 137 | 0.24 | 0.53 |
| PEF1 | 7 | 31 | 284 | 0.24 | 0.55 |
| CMC4 | 3 | 26.5 | 68 | 0.24 | 0.53 |
| CHKA | 6 | 25.3 | 439 | 0.24 | 0.54 |
| RRM1 | 29 | 49 | 792 | 0.24 | 0.41 |
| ACBD3 | 14 | 33.3 | 528 | 0.24 | 0.40 |
| CTNNA1 | 34 | 41.6 | 906 | 0.23 | 0.63 |
| SUCLA2 | 15 | 32.4 | 441 | 0.23 | 0.59 |
| FEN1 | 16 | 48.7 | 380 | 0.23 | 0.41 |
| CCDC134 | 2 | 13.5 | 229 | 0.23 | 0.45 |

| **Table S1.** (Continued) | | | | | |
| --- | --- | --- | --- | --- | --- |
| *Gene(s)* | *Unique*  *Peptides* | *Sequence*  *Coverage (%)* | *Sequence*  *Length* | *Log2*  *(PYR/DMSO)* | *-Log10*  *(P-Value)* |
| KPNA6 | 4 | 23.5 | 536 | 0.24 | 0.74 |
| API5 | 23 | 50.4 | 504 | 0.24 | 0.62 |
| FAM98A | 4 | 17.2 | 518 | 0.24 | 0.55 |
| PRKAB1 | 4 | 27 | 270 | 0.23 | 0.58 |
| KIF20B | 5 | 3.4 | 1722 | 0.23 | 0.44 |
| GTF2I | 13 | 17.3 | 957 | 0.23 | 0.58 |
| RPLP0; RPLP0P6 | 13 | 47 | 317 | 0.23 | 0.67 |
| MAP2K2 | 5 | 30.5 | 400 | 0.23 | 0.79 |
| GOLPH3 | 5 | 22.8 | 298 | 0.23 | 0.75 |
| NIPBL | 6 | 7.4 | 1101 | 0.23 | 0.44 |
| DDX55 | 2 | 4.4 | 569 | 0.23 | 0.82 |
| HSDL2 | 6 | 19.4 | 418 | 0.22 | 0.58 |
| CCNK | 11 | 34.9 | 378 | 0.22 | 0.67 |
| KPNA4 | 9 | 38 | 521 | 0.22 | 0.40 |
| HIST2H3A; HIST3H3; HIST1H3A; H3F3B; H3F3A; HIST2H3PS2; H3F3C | 9 | 40.4 | 136 | 0.22 | 0.56 |
| GNB1 | 3 | 26.5 | 340 | 0.22 | 0.55 |
| DPP7 | 2 | 5.1 | 492 | 0.22 | 0.66 |
| DEF6 | 4 | 6 | 631 | 0.22 | 0.48 |
| APOBEC3C | 3 | 24.2 | 190 | 0.22 | 0.61 |
| MAPK1IP1L | 2 | 10.2 | 245 | 0.22 | 0.51 |
| COMMD10 | 6 | 35.1 | 202 | 0.22 | 0.62 |
| ZWILCH | 3 | 5.4 | 591 | 0.22 | 0.38 |
| ENPP1 | 4 | 7.5 | 925 | 0.22 | 0.53 |
| TNPO1 | 21 | 41.8 | 890 | 0.22 | 0.37 |
| UBE2J1 | 2 | 4.7 | 318 | 0.22 | 0.54 |
| ZBTB1 | 3 | 7.1 | 644 | 0.22 | 0.77 |
| EIF2D | 15 | 34.2 | 584 | 0.22 | 0.36 |
| LONP1 | 32 | 42.4 | 845 | 0.22 | 0.57 |
| PATL1 | 5 | 11.5 | 703 | 0.22 | 0.66 |
| RNMTL1 | 3 | 9.5 | 420 | 0.21 | 0.58 |
| FASTKD2 | 13 | 22.4 | 710 | 0.21 | 0.40 |
| EBNA1BP2 | 12 | 31 | 361 | 0.21 | 0.55 |
| COMMD4 | 4 | 24.6 | 199 | 0.21 | 0.47 |
| UTP18 | 7 | 17.3 | 556 | 0.21 | 0.46 |
| TNFAIP1 | 3 | 15.8 | 316 | 0.21 | 0.75 |
| TCP1 | 32 | 64.2 | 556 | 0.21 | 0.44 |
| ATP5C1 | 2 | 7.7 | 297 | 0.21 | 0.79 |
| RCL1 | 4 | 11.5 | 373 | 0.21 | 0.65 |
| UBE2N; UBE2NL | 8 | 56.6 | 152 | 0.21 | 0.48 |
| BAZ1B | 10 | 7.5 | 1479 | 0.21 | 0.41 |
| RPL34 | 6 | 36.8 | 117 | 0.21 | 0.48 |
| SATB1 | 3 | 7.6 | 763 | 0.21 | 0.43 |
| NOP56 | 3 | 8.4 | 594 | 0.21 | 0.47 |
| COPS2 | 16 | 43.6 | 443 | 0.21 | 0.35 |
| GRN | 6 | 25.3 | 388 | 0.21 | 0.63 |
| SEC22B | 7 | 38.6 | 215 | 0.21 | 0.44 |
| RBM25 | 21 | 28.4 | 843 | 0.21 | 0.50 |
| RCOR3 | 4 | 11.6 | 449 | 0.21 | 0.38 |
| TRAP1 | 20 | 36.5 | 704 | 0.21 | 0.53 |
| CYR61 | 8 | 34.9 | 381 | 0.21 | 0.66 |
| UNC45A | 18 | 21.3 | 944 | 0.21 | 0.35 |
| NECAP2 | 7 | 39.2 | 263 | 0.21 | 0.49 |
| RMND5A | 4 | 14.1 | 391 | 0.21 | 0.51 |
| CAP2 | 10 | 33.1 | 477 | 0.21 | 0.49 |
| VPS28 | 9 | 52.9 | 221 | 0.21 | 0.54 |
| PARN | 16 | 28.5 | 578 | 0.21 | 0.48 |
| COMMD5 | 3 | 21.1 | 171 | 0.21 | 0.70 |
| CDK12 | 4 | 3.2 | 1481 | 0.20 | 0.50 |
| PDSS1 | 2 | 6.1 | 376 | 0.20 | 0.44 |
| CCDC58 | 8 | 69.4 | 144 | 0.20 | 0.42 |
| CCT6A | 28 | 61.6 | 531 | 0.20 | 0.39 |
| CUX1 | 3 | 2.4 | 1403 | 0.20 | 0.43 |
| CAP1 | 29 | 67.2 | 475 | 0.20 | 0.53 |
| ESYT1 | 8 | 10.8 | 1104 | 0.20 | 0.37 |
| FOCAD | 4 | 3.3 | 1237 | 0.20 | 0.36 |
| BRIX1 | 9 | 30.9 | 353 | 0.20 | 0.73 |
| TBCC | 7 | 28.3 | 346 | 0.20 | 0.35 |
| DTYMK | 13 | 57.1 | 212 | 0.20 | 0.62 |
| SETD3 | 7 | 11.3 | 594 | 0.20 | 0.35 |
| GTSE1 | 4 | 5.6 | 720 | 0.20 | 0.51 |
| COPS4 | 21 | 65.3 | 406 | 0.20 | 0.63 |
| EIF3K | 9 | 55.5 | 218 | 0.20 | 0.47 |
| GCLM | 10 | 46.7 | 274 | 0.20 | 0.52 |
| FAM111B | 2 | 3 | 734 | 0.20 | 0.43 |
| RABGEF1 | 8 | 23.6 | 407 | 0.20 | 0.39 |
| ACTR5 | 6 | 12.2 | 607 | 0.20 | 0.61 |
| RPL35 | 4 | 25.2 | 123 | 0.20 | 0.72 |
| CD55 | 4 | 14.8 | 317 | 0.20 | 0.34 |
| SF3A2 | 8 | 21.6 | 464 | 0.20 | 0.66 |
| UBE2Q1; UBE2Q2 | 2 | 3.8 | 422 | 0.20 | 0.41 |
| TMX1 | 5 | 15 | 280 | 0.20 | 0.39 |
| RPL15 | 6 | 26.4 | 174 | 0.20 | 0.40 |
| SUPV3L1 | 9 | 15.9 | 786 | 0.19 | 0.42 |
| CACYBP | 20 | 75.9 | 228 | 0.19 | 0.45 |
| VPS4A | 4 | 20.8 | 437 | 0.19 | 0.49 |
| COPS7B | 4 | 21.7 | 230 | 0.19 | 0.37 |
| RRP7A | 6 | 28.9 | 280 | 0.19 | 0.41 |
| MMP14 | 2 | 3.4 | 582 | 0.19 | 0.34 |
| SIRT2 | 2 | 4.6 | 389 | 0.19 | 0.76 |
| PRKAA1 | 6 | 13.1 | 559 | 0.19 | 0.33 |
| BAZ1A | 2 | 1.3 | 1524 | 0.19 | 0.31 |
| MBD1 | 2 | 14.6 | 240 | 0.19 | 0.43 |
| VAPB | 6 | 25.5 | 243 | 0.19 | 0.36 |
| TMEM87A | 3 | 8.5 | 494 | 0.19 | 0.41 |
| SCAF4 | 8 | 10.2 | 1132 | 0.19 | 0.48 |
| UBE2M | 9 | 47 | 183 | 0.19 | 0.46 |
| CDCA5 | 6 | 32.6 | 307 | 0.19 | 1.01 |
| STAM2 | 15 | 36.4 | 525 | 0.19 | 0.46 |
| DDX5 | 16 | 42.8 | 614 | 0.19 | 0.36 |
| SHC1 | 8 | 22.2 | 473 | 0.19 | 0.76 |
| IMMT | 3 | 9 | 659 | 0.19 | 0.96 |
| TBRG4 | 5 | 6.8 | 631 | 0.19 | 0.67 |
| H2AFX; HIST1H2AJ; HIST1H2AH; H2AFJ; HIST2H2AC; HIST1H2AC; HIST3H2A; HIST2H2AA3; HIST1H2AD; HIST1H2AG; HIST1H2AB; HIST1H2AA; HIST2H2AB | 3 | 21.7 | 143 | 0.19 | 0.39 |
| PARP1 | 51 | 53 | 1014 | 0.19 | 0.45 |
| TK1 | 9 | 48.3 | 234 | 0.19 | 0.44 |
| IPO13 | 3 | 3.5 | 963 | 0.19 | 0.63 |
| BZW2 | 22 | 51.6 | 419 | 0.19 | 0.46 |
| MMAB | 7 | 37.2 | 250 | 0.19 | 0.41 |
| CCT5 | 39 | 80.4 | 541 | 0.19 | 0.47 |
| LMNB2 | 31 | 50.3 | 620 | 0.19 | 0.35 |
| ANXA11 | 13 | 30.5 | 472 | 0.19 | 0.47 |
| SERPINE1 | 5 | 19.7 | 402 | 0.19 | 0.44 |
| GABPA | 12 | 37.7 | 454 | 0.19 | 0.54 |
| PPP3CB | 3 | 14.9 | 524 | 0.19 | 0.35 |
| PDHX | 11 | 46.1 | 486 | 0.19 | 0.44 |
| REEP5 | 4 | 18 | 189 | 0.19 | 0.47 |
| ASCC1 | 5 | 11.5 | 357 | 0.19 | 0.40 |
| POLE2 | 2 | 5.8 | 501 | 0.19 | 0.38 |
| USP47 | 28 | 26.3 | 1355 | 0.19 | 0.48 |
| KIAA0020 | 3 | 3.5 | 648 | 0.18 | 0.45 |
| TOP2A | 4 | 5.4 | 1531 | 0.18 | 0.40 |
| RANGAP1 | 25 | 52.3 | 587 | 0.18 | 0.46 |
| ABR | 7 | 11.3 | 822 | 0.18 | 0.43 |
| INTS10 | 3 | 3.9 | 710 | 0.18 | 0.39 |
| PAK1IP1 | 13 | 40.3 | 392 | 0.18 | 0.33 |
| MED20 | 5 | 28.3 | 212 | 0.18 | 0.46 |
| CDC5L | 12 | 18.5 | 802 | 0.18 | 0.35 |
| STX7 | 8 | 49.4 | 239 | 0.18 | 0.55 |
| GSTK1 | 3 | 23.7 | 190 | 0.18 | 0.58 |
| RNH1 | 24 | 73.5 | 461 | 0.18 | 0.46 |
| SF3A3 | 24 | 54.1 | 501 | 0.18 | 0.43 |
| TFB2M | 11 | 27.3 | 396 | 0.18 | 0.39 |
| EFEMP1 | 2 | 5.4 | 485 | 0.18 | 0.37 |
| SMC6 | 7 | 7.8 | 1091 | 0.18 | 0.45 |
| INTS8 | 3 | 5.6 | 800 | 0.18 | 0.35 |
| PCBP2 | 9 | 55.8 | 362 | 0.18 | 0.34 |
| CCT7 | 32 | 69.6 | 543 | 0.18 | 0.56 |
| RPL38 | 3 | 38.6 | 70 | 0.18 | 0.69 |
| KDM5C | 9 | 6.7 | 1516 | 0.18 | 0.52 |
| GRPEL2 | 6 | 28.9 | 225 | 0.18 | 0.40 |
| ILF2 | 15 | 56.2 | 352 | 0.18 | 1.49 |
| EIF3C; EIF3CL | 29 | 33.2 | 903 | 0.18 | 0.33 |
| UBXN4 | 6 | 22.2 | 508 | 0.18 | 0.53 |
| POLR2G | 8 | 59.3 | 172 | 0.18 | 0.39 |
| PCBP1 | 11 | 67.1 | 356 | 0.18 | 0.46 |
| CCAR2 | 31 | 42.7 | 923 | 0.18 | 0.40 |
| C17orf75 | 5 | 17.4 | 396 | 0.18 | 0.50 |
| PSMD12 | 22 | 53.9 | 456 | 0.18 | 0.44 |
| EXOSC3 | 9 | 46.2 | 275 | 0.18 | 0.38 |
| KPNA3 | 10 | 44.1 | 521 | 0.18 | 0.43 |
| CCNT1 | 8 | 11.3 | 726 | 0.18 | 0.41 |
| U2SURP | 23 | 25.6 | 1028 | 0.18 | 0.37 |
| ZFC3H1 | 6 | 3.7 | 1910 | 0.18 | 0.34 |
| TIMM10B | 2 | 39.8 | 103 | 0.18 | 0.50 |
| CUL4B | 23 | 35.6 | 900 | 0.18 | 0.31 |
| ECHDC1 | 6 | 30.3 | 284 | 0.18 | 0.32 |
| ARFGEF2 | 3 | 2.7 | 1785 | 0.18 | 0.37 |
| COA4 | 6 | 75.9 | 87 | 0.18 | 0.80 |
| KATNA1 | 2 | 4.1 | 491 | 0.18 | 0.43 |
| KLHL11 | 2 | 3 | 708 | 0.18 | 0.38 |
| AAGAB | 4 | 12.7 | 315 | 0.18 | 0.37 |
| ORC6 | 3 | 14.8 | 203 | 0.18 | 0.73 |
| ARHGEF1 | 6 | 8.3 | 968 | 0.18 | 0.41 |
| MFI2 | 2 | 8.9 | 302 | 0.18 | 0.30 |
| AP3S1 | 5 | 26.4 | 193 | 0.18 | 0.29 |
| PWP2 | 3 | 4.9 | 919 | 0.18 | 1.07 |
| ARID2 | 2 | 1.3 | 1445 | 0.18 | 0.60 |
| LYAR | 13 | 47 | 379 | 0.18 | 0.42 |
| EIF3M | 17 | 57.8 | 374 | 0.18 | 0.31 |
| PROCR | 4 | 21.6 | 213 | 0.18 | 0.67 |
| COPZ1 | 6 | 37.4 | 198 | 0.18 | 0.74 |
| SNX1 | 13 | 38.1 | 457 | 0.17 | 0.42 |
| KIF2C | 7 | 11.3 | 725 | 0.17 | 0.41 |
| VIM | 32 | 72.3 | 466 | 0.17 | 0.32 |
| SNX2 | 14 | 34.7 | 519 | 0.17 | 0.32 |
| FUT11 | 2 | 8 | 476 | 0.17 | 0.35 |
| PSMD13 | 21 | 66.2 | 376 | 0.17 | 0.54 |
| RPL10A | 10 | 44.2 | 217 | 0.17 | 0.43 |
| EIF2S2 | 25 | 66.7 | 333 | 0.17 | 0.43 |
| PSME3 | 14 | 66.1 | 254 | 0.17 | 0.34 |
| PLS1 | 26 | 52.1 | 629 | 0.17 | 0.30 |
| RAD18 | 6 | 12.7 | 495 | 0.17 | 0.35 |
| ABL1 | 3 | 4.1 | 1130 | 0.17 | 0.38 |
| KPNA1 | 7 | 33.1 | 538 | 0.17 | 0.38 |
| RFC2 | 2 | 4.4 | 320 | 0.17 | 0.55 |
| AASDHPPT | 12 | 39.8 | 309 | 0.17 | 0.43 |
| PICALM | 6 | 14 | 551 | 0.17 | 0.41 |
| SMARCD1 | 6 | 18.6 | 515 | 0.17 | 0.37 |
| MYH15 | 8 | 4.9 | 1946 | 0.17 | 0.31 |
| CCT3 | 35 | 65.7 | 545 | 0.17 | 0.29 |
| PVRL2 | 4 | 8.1 | 479 | 0.17 | 0.51 |
| TOR1AIP2 | 10 | 31.1 | 470 | 0.17 | 0.36 |
| MRPL47 | 2 | 8.3 | 230 | 0.17 | 0.38 |
| NDUFAF4 | 4 | 18.9 | 175 | 0.17 | 0.51 |
| PRPF19 | 18 | 55.8 | 504 | 0.17 | 0.36 |
| NCOA5 | 14 | 35.1 | 579 | 0.17 | 0.38 |
| KNOP1 | 7 | 22.1 | 458 | 0.17 | 0.38 |
| FAM114A2 | 3 | 11.1 | 505 | 0.17 | 0.44 |
| MRPS25 | 3 | 32.4 | 173 | 0.17 | 0.51 |
| RPA2 | 7 | 56.3 | 270 | 0.17 | 0.45 |
| RBM15 | 7 | 8.4 | 933 | 0.17 | 0.88 |
| RNF168 | 2 | 3.2 | 571 | 0.17 | 0.40 |
| NPM1 | 11 | 65 | 294 | 0.17 | 0.33 |
| POLR3F | 6 | 21.5 | 316 | 0.17 | 0.37 |
| QSOX1 | 5 | 9.3 | 604 | 0.17 | 0.36 |
| WDR3 | 3 | 4.1 | 943 | 0.17 | 0.39 |
| HSD17B4 | 35 | 59.8 | 736 | 0.17 | 0.33 |
| ALDH18A1 | 32 | 47.5 | 793 | 0.17 | 0.37 |
| AP1M1 | 13 | 43.6 | 351 | 0.17 | 0.39 |
| RSL24D1 | 2 | 16.6 | 163 | 0.17 | 0.53 |
| ROCK2 | 32 | 27.8 | 1388 | 0.17 | 0.37 |
| PSMD8 | 12 | 57.1 | 287 | 0.17 | 0.46 |
| ARL6IP1 | 3 | 21.5 | 237 | 0.17 | 0.68 |
| TUBA4A | 3 | 57 | 433 | 0.17 | 0.43 |
| PSMD11 | 28 | 72.7 | 422 | 0.17 | 0.44 |
| TMEM57 | 2 | 6.5 | 306 | 0.17 | 0.35 |
| C18orf8 | 11 | 23.3 | 657 | 0.17 | 0.29 |
| PSMD14 | 15 | 53.2 | 310 | 0.17 | 0.34 |
| FXR1 | 19 | 42.2 | 621 | 0.17 | 0.42 |
| CCT2 | 35 | 80.6 | 535 | 0.17 | 0.28 |
| DNAJA1 | 19 | 55.7 | 397 | 0.17 | 0.48 |
| FERMT2 | 10 | 20 | 640 | 0.16 | 0.31 |
| DBT | 7 | 22.4 | 482 | 0.16 | 0.36 |
| SF3B1 | 34 | 33.1 | 1304 | 0.16 | 0.32 |
| NOP2 | 17 | 26.5 | 808 | 0.16 | 0.37 |
| UTP15 | 8 | 21.1 | 545 | 0.16 | 0.35 |
| GSK3A | 2 | 13.3 | 483 | 0.16 | 0.27 |
| GPT2 | 4 | 10.1 | 523 | 0.16 | 0.41 |
| BAG5 | 10 | 29.5 | 447 | 0.16 | 0.35 |
| MBD3 | 4 | 25.5 | 235 | 0.16 | 0.47 |
| ARIH1 | 10 | 24.8 | 557 | 0.16 | 0.39 |
| FLAD1 | 9 | 24.2 | 446 | 0.16 | 0.49 |
| CDKN2AIP | 4 | 9.3 | 580 | 0.16 | 0.36 |
| PELP1 | 18 | 25.4 | 1130 | 0.16 | 0.39 |
| NUDC | 26 | 68.6 | 331 | 0.16 | 0.40 |
| IDH3A | 10 | 31.4 | 331 | 0.16 | 0.42 |
| PRKCA | 7 | 11.9 | 672 | 0.16 | 0.38 |
| ITGB1 | 16 | 20.2 | 798 | 0.16 | 0.36 |
| LYN | 3 | 7.3 | 491 | 0.16 | 0.36 |
| STC2 | 4 | 25.8 | 302 | 0.16 | 0.29 |
| KIF3B | 2 | 6.4 | 373 | 0.16 | 0.38 |
| RNASEH2A | 9 | 32.4 | 299 | 0.16 | 0.37 |
| TRIM24 | 5 | 8.9 | 1016 | 0.16 | 0.43 |
| CCNH | 9 | 31.9 | 323 | 0.16 | 0.28 |
| PTK2 | 13 | 17 | 1017 | 0.16 | 0.38 |
| PDIA6 | 26 | 61.8 | 437 | 0.16 | 0.34 |
| TRIM33 | 7 | 8.9 | 1127 | 0.16 | 0.37 |
| SF3A1 | 30 | 44.8 | 793 | 0.16 | 0.42 |
| HGS | 15 | 27.4 | 777 | 0.16 | 0.41 |
| UFC1 | 6 | 26.9 | 167 | 0.16 | 0.42 |
| TRAF2 | 11 | 26.7 | 490 | 0.16 | 0.36 |
| TFRC | 20 | 29.5 | 760 | 0.16 | 0.32 |
| PRPF38A | 8 | 21.8 | 312 | 0.16 | 0.39 |
| YTHDF2 | 14 | 35.9 | 579 | 0.16 | 0.36 |
| OGT | 11 | 14.2 | 1046 | 0.16 | 0.27 |
| OGFOD2 | 2 | 8.3 | 290 | 0.16 | 0.38 |
| HNRNPM | 17 | 27.4 | 691 | 0.16 | 0.30 |
| CCT8 | 43 | 82.8 | 548 | 0.16 | 0.28 |
| PHC2 | 4 | 10.3 | 464 | 0.16 | 0.61 |
| SMARCA4 | 7 | 7.9 | 1679 | 0.16 | 0.37 |
| PRDX4 | 11 | 59 | 271 | 0.16 | 0.30 |
| ZMYND8 | 18 | 19.9 | 1135 | 0.16 | 0.31 |
| AK3 | 17 | 68.3 | 227 | 0.16 | 0.37 |
| ITGA3 | 4 | 3.8 | 1051 | 0.16 | 0.41 |
| DIABLO | 9 | 52.7 | 186 | 0.16 | 0.41 |
| DPP8 | 3 | 3.5 | 849 | 0.16 | 0.41 |
| YME1L1 | 4 | 8.2 | 683 | 0.16 | 0.25 |
| SLC4A2 | 5 | 8.6 | 1227 | 0.16 | 0.32 |
| TWISTNB | 6 | 24.6 | 338 | 0.16 | 0.35 |
| BUB1 | 4 | 4.5 | 1028 | 0.16 | 0.53 |
| ARMC1 | 8 | 36.9 | 282 | 0.16 | 0.36 |
| AMFR | 2 | 16.1 | 299 | 0.16 | 0.33 |
| PHB | 4 | 19.5 | 272 | 0.16 | 0.35 |
| PRPF18 | 4 | 18.9 | 249 | 0.16 | 0.48 |
| SLC30A1 | 2 | 5.7 | 507 | 0.16 | 0.32 |
| RTN3 | 4 | 7.4 | 1013 | 0.16 | 0.37 |
| CRNKL1 | 18 | 24 | 836 | 0.16 | 0.39 |
| NSUN4 | 2 | 6.6 | 335 | 0.16 | 0.32 |
| ALDH3A2 | 4 | 11.7 | 393 | 0.16 | 0.38 |
| RPA1 | 24 | 48.2 | 616 | 0.16 | 0.34 |
| GLRX3 | 16 | 56.7 | 335 | 0.16 | 0.40 |
| COMMD9 | 8 | 57.6 | 198 | 0.16 | 0.40 |
| MTA1 | 5 | 10.5 | 703 | 0.16 | 0.74 |
| LIG3 | 6 | 8.5 | 862 | 0.16 | 0.36 |
| ADD3 | 6 | 14.5 | 674 | 0.16 | 0.34 |
| TRIM28 | 33 | 61.9 | 835 | 0.16 | 0.39 |
| PRPF40A | 20 | 23.4 | 930 | 0.16 | 0.35 |
| MAEA | 4 | 24 | 183 | 0.16 | 0.88 |
| NOC3L | 4 | 5.6 | 800 | 0.16 | 0.33 |
| STRN4 | 3 | 10 | 753 | 0.16 | 0.28 |
| COPS6 | 8 | 31 | 326 | 0.16 | 0.47 |
| GNA11 | 5 | 17 | 359 | 0.16 | 0.35 |
| NOP14 | 6 | 8.4 | 788 | 0.15 | 0.72 |
| RMDN3 | 5 | 18.3 | 306 | 0.15 | 0.26 |
| ENG | 3 | 6.9 | 625 | 0.15 | 0.30 |
| NOB1 | 3 | 7 | 412 | 0.15 | 0.29 |
| NCKAP1 | 16 | 16.4 | 1128 | 0.15 | 0.25 |
| EIF4A3 | 21 | 62.3 | 411 | 0.15 | 0.31 |
| ITPA | 10 | 72.2 | 194 | 0.15 | 0.43 |
| RBBP7 | 9 | 47.1 | 425 | 0.15 | 0.31 |
| MAPK1 | 14 | 60.6 | 360 | 0.15 | 0.35 |
| EMD | 8 | 36.6 | 254 | 0.15 | 0.33 |
| DNAJB12 | 2 | 4.6 | 409 | 0.15 | 0.27 |
| EIF4E2 | 3 | 16.9 | 213 | 0.15 | 0.59 |
| EPHA2 | 8 | 13.2 | 976 | 0.15 | 0.33 |
| EMC8 | 2 | 12.4 | 210 | 0.15 | 0.26 |
| RPL18 | 7 | 37.8 | 164 | 0.15 | 0.56 |
| MRPS9 | 5 | 14.6 | 396 | 0.15 | 0.37 |
| CHCHD1 | 2 | 22.9 | 118 | 0.15 | 0.34 |
| POLG2 | 7 | 17.3 | 485 | 0.15 | 0.31 |
| IGF2R | 17 | 8 | 2491 | 0.15 | 0.26 |
| LYPLA2 | 7 | 33.8 | 231 | 0.15 | 0.33 |
| NUDCD3 | 12 | 35.5 | 361 | 0.15 | 0.40 |
| GPN1 | 6 | 24.1 | 374 | 0.15 | 0.30 |
| SMAP1 | 6 | 18.1 | 436 | 0.15 | 0.32 |
| PDCD10 | 7 | 46.2 | 186 | 0.15 | 0.57 |
| D2HGDH | 3 | 7.9 | 521 | 0.15 | 0.32 |
| HPCAL1; HPCA | 14 | 75.1 | 193 | 0.15 | 0.42 |
| RBM12 | 20 | 21.8 | 932 | 0.15 | 0.38 |
| COPB2 | 43 | 58.2 | 877 | 0.15 | 0.31 |
| ENGASE | 3 | 5.8 | 743 | 0.15 | 0.29 |
| MRPL52 | 2 | 47.1 | 51 | 0.15 | 0.33 |
| INTS7 | 2 | 4.4 | 913 | 0.15 | 0.45 |
| TBC1D22A | 4 | 10.1 | 487 | 0.15 | 1.69 |
| BABAM1 | 6 | 28.6 | 329 | 0.15 | 0.36 |
| RRBP1 | 50 | 51.9 | 1410 | 0.15 | 0.55 |
| GSK3B | 6 | 30.5 | 420 | 0.15 | 0.25 |
| CC2D1A | 11 | 16.5 | 950 | 0.15 | 0.37 |
| SYMPK | 15 | 12.5 | 1274 | 0.15 | 0.34 |
| ZCCHC17 | 2 | 15.8 | 146 | 0.15 | 1.00 |
| LMNB1 | 33 | 63.7 | 586 | 0.15 | 0.25 |
| NUP50 | 24 | 61.5 | 468 | 0.15 | 0.38 |
| VCL | 82 | 75.4 | 1066 | 0.15 | 0.36 |
| UTP3 | 7 | 17.3 | 479 | 0.15 | 0.31 |
| SMARCC1 | 21 | 27.7 | 1105 | 0.15 | 0.34 |
| HNRNPL | 9 | 42.3 | 589 | 0.15 | 0.26 |
| SNRNP200 | 63 | 35.6 | 2136 | 0.15 | 0.32 |
| THADA | 6 | 4 | 1953 | 0.15 | 0.33 |
| LCP1 | 36 | 72.7 | 627 | 0.15 | 0.35 |
| RAI14 | 11 | 14.5 | 951 | 0.15 | 0.29 |
| AURKA | 8 | 21.3 | 403 | 0.15 | 0.40 |
| MTMR9 | 4 | 11.1 | 549 | 0.15 | 0.28 |
| STK24 | 9 | 47.1 | 412 | 0.15 | 0.42 |
| TOM1L1 | 5 | 22.1 | 326 | 0.15 | 0.41 |
| SLTM | 21 | 29.6 | 1034 | 0.15 | 0.30 |
| PPP2R1B | 4 | 22.3 | 556 | 0.15 | 0.34 |
| SAMD1 | 3 | 10.2 | 432 | 0.15 | 0.74 |
| COIL | 4 | 9.5 | 576 | 0.15 | 0.27 |
| RPRD1B | 14 | 55.2 | 326 | 0.15 | 0.30 |
| SDAD1 | 3 | 4.9 | 650 | 0.15 | 0.50 |
| PSAP | 23 | 47.6 | 527 | 0.15 | 0.63 |
| CHERP | 15 | 16.9 | 927 | 0.14 | 0.30 |
| RPAP3 | 26 | 42.4 | 665 | 0.14 | 0.41 |
| NIFK | 11 | 46.8 | 293 | 0.14 | 0.29 |
| LAMP2 | 3 | 7.1 | 410 | 0.14 | 0.29 |
| MRPS26 | 3 | 8.8 | 205 | 0.14 | 0.27 |
| IRF2BPL | 10 | 19.8 | 796 | 0.14 | 0.35 |
| AGO2; AGO1; AGO3; AGO4 | 2 | 2.2 | 825 | 0.14 | 0.26 |
| RPL12 | 11 | 71.5 | 165 | 0.14 | 0.40 |
| DCXR | 7 | 36.5 | 244 | 0.14 | 0.38 |
| NIP7 | 10 | 76.7 | 180 | 0.14 | 0.24 |
| PRPF8 | 64 | 34 | 2335 | 0.14 | 0.32 |
| COMMD8 | 5 | 35 | 183 | 0.14 | 0.35 |
| BRD4 | 14 | 15.2 | 1362 | 0.14 | 0.33 |
| SWAP70 | 24 | 48.2 | 585 | 0.14 | 0.41 |
| CHCHD4 | 8 | 52.8 | 142 | 0.14 | 0.25 |
| POM121C; POM121; POMZP3 | 4 | 6.6 | 987 | 0.14 | 0.74 |
| GNAI1 | 2 | 19.8 | 354 | 0.14 | 0.26 |
| ZCRB1 | 2 | 14.7 | 217 | 0.14 | 0.27 |
| CHD4 | 13 | 8.9 | 1905 | 0.14 | 1.11 |
| NHP2L1 | 8 | 53.1 | 128 | 0.14 | 0.23 |
| PSMD5 | 17 | 43 | 461 | 0.14 | 0.34 |
| RPS4X | 13 | 46.8 | 263 | 0.14 | 0.76 |
| MATR3 | 20 | 29.4 | 847 | 0.14 | 0.63 |
| TPBG | 4 | 7.6 | 420 | 0.14 | 0.32 |
| NOC2L | 12 | 14.4 | 749 | 0.14 | 0.31 |
| UBR4 | 53 | 11.6 | 5159 | 0.14 | 0.35 |
| RPP14 | 5 | 47.6 | 124 | 0.14 | 0.25 |
| PRKAR2A | 16 | 45.3 | 404 | 0.14 | 0.33 |
| CANX | 16 | 27.4 | 592 | 0.14 | 0.32 |
| LEPREL2 | 6 | 14.7 | 736 | 0.14 | 0.32 |
| OSGEP | 9 | 36.4 | 335 | 0.14 | 0.24 |
| MGME1 | 13 | 41.6 | 344 | 0.14 | 0.34 |
| PHF2 | 3 | 3.1 | 1096 | 0.14 | 0.25 |
| GMPS | 36 | 62.5 | 693 | 0.14 | 0.30 |
| ANAPC2 | 4 | 5.1 | 819 | 0.14 | 0.25 |
| MAK16 | 7 | 31.7 | 300 | 0.14 | 0.34 |
| TIPRL | 11 | 40.4 | 272 | 0.14 | 0.29 |
| LAGE3 | 2 | 21 | 143 | 0.14 | 0.63 |
| ATP6V1E1 | 13 | 56.6 | 226 | 0.14 | 0.32 |
| PLEC | 2 | 54.8 | 4547 | 0.14 | 0.33 |
| TOMM22 | 2 | 32.4 | 142 | 0.14 | 0.33 |
| VPS4B | 7 | 27.7 | 444 | 0.14 | 0.34 |
| SCAMP3 | 7 | 38.3 | 347 | 0.14 | 0.38 |
| KHDRBS1; KHDRBS2 | 9 | 22.5 | 418 | 0.14 | 0.32 |
| GPBP1L1 | 2 | 5.5 | 474 | 0.14 | 0.28 |
| UBE2O | 32 | 36.7 | 1292 | 0.14 | 0.23 |
| ANLN | 30 | 31.1 | 1124 | 0.14 | 0.35 |
| NCBP1 | 22 | 37.2 | 790 | 0.14 | 0.32 |
| PSME1 | 12 | 54.2 | 249 | 0.14 | 0.37 |
| SLC39A14 | 2 | 4.2 | 481 | 0.14 | 0.34 |
| KRI1 | 13 | 24 | 703 | 0.14 | 0.26 |
| OSBP | 10 | 17.3 | 807 | 0.14 | 0.31 |
| KPNB1 | 36 | 51 | 876 | 0.14 | 0.33 |
| HELLS | 3 | 4.9 | 884 | 0.14 | 0.60 |
| NOL11 | 19 | 33.9 | 719 | 0.14 | 0.24 |
| BRD2 | 10 | 20.9 | 807 | 0.14 | 0.31 |
| KRR1 | 7 | 20.7 | 381 | 0.14 | 0.34 |
| ARAP1 | 4 | 4.1 | 1439 | 0.14 | 0.26 |
| CLPP | 9 | 41.9 | 277 | 0.14 | 0.33 |
| KDM1A | 17 | 29.2 | 852 | 0.14 | 0.28 |
| FAM91A1 | 4 | 7.1 | 789 | 0.14 | 0.29 |
| SAP18 | 8 | 48.8 | 172 | 0.14 | 0.26 |
| USP9X | 21 | 18.7 | 2554 | 0.14 | 0.32 |
| CGGBP1 | 6 | 42.5 | 167 | 0.14 | 0.34 |
| GIT2 | 4 | 8 | 641 | 0.14 | 0.37 |
| NAPA | 13 | 59 | 295 | 0.14 | 0.28 |
| ITGAV | 3 | 3.6 | 1002 | 0.14 | 0.28 |
| MRPL42 | 3 | 34.4 | 128 | 0.14 | 0.28 |
| NRP2 | 2 | 3.8 | 901 | 0.14 | 0.93 |
| FIBP | 4 | 11.8 | 357 | 0.14 | 0.42 |
| GGA3 | 5 | 8.6 | 690 | 0.14 | 0.29 |
| SF3B6 | 4 | 39.2 | 125 | 0.14 | 0.26 |
| DNAJC3 | 19 | 38.3 | 504 | 0.14 | 0.27 |
| CPD | 6 | 5.1 | 1380 | 0.14 | 0.29 |
| RSL1D1 | 16 | 38.8 | 490 | 0.14 | 0.28 |
| C11orf68 | 6 | 21.2 | 292 | 0.14 | 0.37 |
| RBM14 | 12 | 28.6 | 669 | 0.14 | 0.28 |
| CFAP20 | 5 | 22.8 | 193 | 0.14 | 0.27 |
| GSS | 26 | 61.6 | 474 | 0.14 | 0.25 |
| PTPRE | 4 | 12.4 | 615 | 0.13 | 0.28 |
| COL4A3BP | 7 | 11 | 598 | 0.13 | 0.24 |
| ATP6V1C1 | 21 | 54.2 | 382 | 0.13 | 0.30 |
| ARHGAP12 | 2 | 3.1 | 799 | 0.13 | 0.29 |
| POLR1B | 19 | 18.7 | 1135 | 0.13 | 0.27 |
| PPP6R2 | 2 | 3.1 | 905 | 0.13 | 0.35 |
| NOL8 | 11 | 9.5 | 1099 | 0.13 | 0.23 |
| RPS3 | 15 | 65 | 243 | 0.13 | 0.43 |
| TRMT61B | 5 | 17.8 | 477 | 0.13 | 0.27 |
| UBAC1 | 12 | 35.8 | 405 | 0.13 | 0.29 |
| UBE2T | 9 | 52.8 | 197 | 0.13 | 0.31 |
| RPA3 | 8 | 86.8 | 121 | 0.13 | 0.33 |
| IFI16 | 9 | 15.8 | 673 | 0.13 | 0.25 |
| TTC4 | 8 | 23.3 | 387 | 0.13 | 0.25 |
| PSMF1 | 4 | 21.3 | 183 | 0.13 | 0.26 |
| DHRS4; DHRS4L1 | 2 | 8 | 226 | 0.13 | 0.25 |
| CAMSAP1 | 10 | 5.9 | 1602 | 0.13 | 0.31 |
| NMD3 | 4 | 10.2 | 529 | 0.13 | 0.29 |
| ATG3 | 7 | 26.4 | 314 | 0.13 | 0.24 |
| XPO4 | 15 | 13.4 | 1151 | 0.13 | 0.38 |
| UTRN | 17 | 5 | 3433 | 0.13 | 0.27 |
| SMARCC2 | 22 | 31.1 | 1214 | 0.13 | 0.25 |

**Table S2.** Top 500 proteins destabilized by pyrimethamine in U3A human fibrosarcoma cells.

| *Gene(s)* | *Unique*  *Peptides* | *Sequence*  *Coverage (%)* | *Sequence*  *Length* | *Log2*  *(PYR/DMSO)* | *-Log10*  *(P-Value)* |
| --- | --- | --- | --- | --- | --- |
| RALGAPA1 | 3 | 1.2 | 2495 | -0.50 | 2.00 |
| RHEB | 5 | 64.6 | 79 | -0.45 | 1.00 |
| CEP83 | 5 | 12.2 | 352 | -0.43 | 1.88 |
| ANXA8L1; ANXA8L2; ANXA8 | 2 | 11.1 | 270 | -0.40 | 2.55 |
| FAM162A | 2 | 19.4 | 144 | -0.37 | 1.14 |
| KIAA1598 | 2 | 4 | 498 | -0.28 | 0.91 |
| GAMT | 2 | 22.5 | 213 | -0.27 | 1.02 |
| MGRN1 | 2 | 5.4 | 594 | -0.25 | 0.61 |
| CEP85L | 2 | 2.1 | 805 | -0.24 | 0.78 |
| CNOT7 | 2 | 10.1 | 208 | -0.24 | 0.41 |
| GOLM1 | 10 | 28.9 | 391 | -0.22 | 0.44 |
| PNKP | 2 | 5.6 | 485 | -0.22 | 0.72 |
| CEP192 | 2 | 1.2 | 2062 | -0.22 | 3.06 |
| ING1 | 2 | 5 | 422 | -0.22 | 0.83 |
| CDK5RAP2 | 3 | 1.6 | 1663 | -0.21 | 0.86 |
| GAK | 3 | 7.9 | 416 | -0.20 | 0.95 |
| PRSS1; PRSS3P2; PRSS2 | 2 | 21.1 | 142 | -0.20 | 0.70 |
| CEP97 | 2 | 3 | 806 | -0.20 | 1.26 |
| R3HCC1L | 2 | 6.2 | 792 | -0.20 | 0.57 |
| SERPINB7 | 3 | 12.7 | 363 | -0.19 | 0.38 |
| SMN1; SMN2 | 7 | 28.7 | 282 | -0.18 | 0.55 |
| EIF5A2 | 2 | 66.7 | 153 | -0.18 | 0.36 |
| SNX16 | 2 | 9.2 | 315 | -0.18 | 0.45 |
| ENO1 | 2 | 87.1 | 341 | -0.17 | 0.59 |
| MSI2 | 2 | 11.3 | 328 | -0.17 | 0.43 |
| LSM14B | 3 | 14.3 | 385 | -0.17 | 0.32 |
| RBCK1 | 5 | 18.4 | 468 | -0.16 | 0.30 |
| IFT22 | 2 | 21.1 | 185 | -0.16 | 0.31 |
| DSCR3 | 2 | 12.4 | 249 | -0.16 | 1.73 |
| S100A4 | 3 | 25.7 | 101 | -0.16 | 0.34 |
| ZNF143 | 3 | 4.6 | 610 | -0.16 | 0.90 |
| KIAA1143 | 12 | 64.9 | 154 | -0.16 | 0.25 |
| IQCB1 | 2 | 4.7 | 465 | -0.16 | 0.37 |
| WDR87 | 2 | 0.7 | 2912 | -0.16 | 0.85 |
| PHF14 | 2 | 2 | 948 | -0.15 | 0.37 |
| RAC2 | 4 | 48.4 | 192 | -0.15 | 0.62 |
| SSFA2 | 4 | 4.4 | 1237 | -0.15 | 0.87 |
| PYCRL | 4 | 20.6 | 286 | -0.15 | 1.40 |
| TSEN15 | 2 | 19.9 | 171 | -0.15 | 0.33 |
| CTNNBIP1 | 2 | 29.6 | 81 | -0.15 | 0.22 |
| ARMC9 | 2 | 24.1 | 166 | -0.15 | 0.53 |
| ECM1 | 5 | 12 | 540 | -0.14 | 0.30 |
| HMGB2 | 11 | 58.4 | 209 | -0.14 | 0.22 |
| EIF1AD | 7 | 69.9 | 113 | -0.14 | 1.00 |
| PPIA | 2 | 90.5 | 105 | -0.14 | 0.25 |
| SH3D19 | 4 | 4.1 | 1047 | -0.14 | 1.56 |
| ARFGAP1 | 6 | 25.9 | 340 | -0.14 | 0.78 |
| GUK1 | 3 | 17.1 | 199 | -0.14 | 2.30 |

| **Table S2.** (Continued) | | | | | |
| --- | --- | --- | --- | --- | --- |
| *Gene(s)* | *Unique*  *Peptides* | *Sequence*  *Coverage (%)* | *Sequence*  *Length* | *Log2*  *(PYR/DMSO)* | *-Log10*  *(P-Value)* |
| SYNJ2 | 2 | 2.5 | 1259 | -0.14 | 0.60 |
| OARD1 | 5 | 38.2 | 131 | -0.14 | 0.69 |
| EEF2K | 5 | 6.9 | 725 | -0.14 | 0.38 |
| GOLIM4 | 5 | 10 | 668 | -0.14 | 0.58 |
| GTF2A2 | 2 | 20.2 | 109 | -0.14 | 0.36 |
| PSMG4 | 5 | 52.8 | 123 | -0.13 | 0.20 |
| RBM38; RBM24 | 3 | 35.8 | 123 | -0.13 | 1.23 |
| AGAP1; AGAP3 | 2 | 6.8 | 351 | -0.13 | 0.83 |
| SH3BP5L | 7 | 20.6 | 393 | -0.13 | 0.27 |
| HMGB3 | 10 | 55.5 | 200 | -0.13 | 0.21 |
| ANXA6 | 2 | 64.8 | 460 | -0.13 | 1.17 |
| ERI3 | 4 | 29 | 176 | -0.13 | 0.54 |
| ZSCAN26; ZNF187 | 2 | 7.6 | 344 | -0.13 | 0.58 |
| TEX11 | 2 | 2.6 | 615 | -0.13 | 0.30 |
| POLE4 | 6 | 81.2 | 117 | -0.13 | 0.30 |
| MZT2B; MZT2A | 4 | 29.4 | 218 | -0.13 | 1.56 |
| AKR1B10 | 4 | 28.2 | 316 | -0.13 | 0.26 |
| MPLKIP | 2 | 19 | 179 | -0.13 | 0.38 |
| MYPN | 3 | 4 | 1026 | -0.13 | 0.69 |
| ACTR8 | 3 | 6.9 | 624 | -0.12 | 0.24 |
| MOCS2 | 5 | 53.4 | 88 | -0.12 | 0.28 |
| DNAJB2 | 2 | 14.9 | 228 | -0.12 | 0.54 |
| ATHL1 | 3 | 6.3 | 764 | -0.12 | 1.03 |
| PDC | 2 | 6.9 | 246 | -0.12 | 0.20 |
| FAM207A | 2 | 12.8 | 203 | -0.12 | 0.41 |
| APEH | 25 | 47.9 | 737 | -0.12 | 2.72 |
| SAR1A; SAR1B | 2 | 9.6 | 198 | -0.12 | 0.28 |
| KRT81; KRT86; KRT83; KRT87P; KRT85 | 2 | 7.6 | 484 | -0.12 | 1.00 |
| QSER1 | 2 | 1.5 | 1529 | -0.12 | 0.22 |
| LAS1L | 4 | 6.9 | 734 | -0.12 | 0.25 |
| HCLS1 | 3 | 6.2 | 449 | -0.12 | 0.78 |
| CDKN1B | 3 | 23.7 | 198 | -0.12 | 0.24 |
| ANP32E | 10 | 54.1 | 268 | -0.12 | 0.21 |
| MTPN | 6 | 55.9 | 118 | -0.12 | 0.18 |
| SCOC | 3 | 78 | 82 | -0.11 | 1.35 |
| CFDP1 | 2 | 46.1 | 217 | -0.11 | 0.21 |
| SORBS1 | 2 | 5.1 | 507 | -0.11 | 0.31 |
| CKS2 | 2 | 32.9 | 79 | -0.11 | 0.20 |
| TXNRD1 | 6 | 69.7 | 568 | -0.11 | 1.43 |
| FKBP1A | 4 | 40.7 | 108 | -0.11 | 0.17 |
| SEPT8 | 6 | 27.2 | 430 | -0.10 | 0.54 |
| ACBD7 | 2 | 23.9 | 88 | -0.10 | 0.21 |
| ACY1; ABHD14A-ACY1 | 13 | 46.6 | 373 | -0.10 | 2.05 |
| PHPT1 | 9 | 82.4 | 125 | -0.10 | 0.19 |
| SRP19 | 9 | 63.9 | 144 | -0.10 | 0.16 |
| FOXN3 | 3 | 9.4 | 468 | -0.10 | 0.22 |
| ERCC6L | 13 | 15.3 | 1127 | -0.10 | 0.62 |
| CROCC | 3 | 2 | 1311 | -0.10 | 1.19 |
| PCNT | 2 | 0.7 | 3336 | -0.10 | 0.25 |
| PTPRK | 5 | 5.6 | 1458 | -0.10 | 0.17 |
| TUFM | 23 | 61.9 | 452 | -0.10 | 0.30 |
| INO80B; INO80B-WBP1 | 2 | 13 | 184 | -0.10 | 0.75 |
| ZNF286A; ZNF286B | 2 | 17.9 | 106 | -0.10 | 0.27 |
| SERPINB6 | 20 | 65.8 | 380 | -0.10 | 0.78 |
| CALB2 | 15 | 58.3 | 271 | -0.10 | 0.17 |
| ATE1 | 2 | 54.8 | 511 | -0.10 | 1.63 |
| SERGEF | 7 | 22.5 | 458 | -0.10 | 0.17 |
| SHANK2 | 7 | 7.8 | 1253 | -0.10 | 1.24 |
| LETM2 | 3 | 10.8 | 443 | -0.10 | 0.37 |
| SRP14 | 9 | 50.7 | 136 | -0.10 | 0.15 |
| ARHGAP18 | 3 | 6.5 | 618 | -0.10 | 0.18 |
| ZFAND2B | 2 | 15 | 213 | -0.10 | 0.73 |
| EIF5A; EIF5AL1 | 8 | 85.1 | 154 | -0.10 | 0.18 |
| OSTF1 | 6 | 43 | 214 | -0.10 | 0.16 |
| RPS21 | 7 | 76.5 | 81 | -0.09 | 0.19 |
| CALM2; CALM1; CALM3 | 8 | 90.6 | 149 | -0.09 | 0.14 |
| UTP6 | 22 | 35 | 597 | -0.09 | 0.15 |
| CDKN2C | 7 | 54.8 | 168 | -0.09 | 0.15 |
| NUDT2 | 5 | 53.1 | 147 | -0.09 | 0.18 |
| ITGB1BP1 | 2 | 36.9 | 65 | -0.09 | 0.79 |
| COA7 | 14 | 71.9 | 231 | -0.09 | 0.14 |
| SNX11 | 2 | 9.1 | 254 | -0.09 | 0.15 |
| FAM103A1 | 6 | 44.9 | 118 | -0.09 | 0.16 |
| TNIP1 | 12 | 20.5 | 619 | -0.09 | 1.04 |
| CCDC23 | 3 | 45.5 | 66 | -0.09 | 0.15 |
| ARHGAP31 | 3 | 2.7 | 1444 | -0.09 | 0.22 |
| TPK1 | 6 | 50 | 166 | -0.09 | 1.65 |
| NAT1 | 3 | 10.5 | 352 | -0.09 | 1.20 |
| CARHSP1 | 6 | 75 | 124 | -0.09 | 0.18 |
| DAP | 7 | 59.8 | 102 | -0.09 | 0.14 |
| GATB; PET112 | 5 | 12.4 | 557 | -0.09 | 0.22 |
| MZT1 | 3 | 52.4 | 82 | -0.09 | 0.16 |
| MTIF2 | 6 | 10 | 727 | -0.09 | 0.22 |
| LRRC16A | 2 | 2 | 932 | -0.09 | 0.52 |
| PGP | 11 | 39.6 | 321 | -0.09 | 0.73 |
| GPX1 | 8 | 53 | 202 | -0.09 | 1.31 |
| FNBP1L | 2 | 4.7 | 451 | -0.09 | 0.36 |
| TRPV1; SHPK | 10 | 33.5 | 478 | -0.09 | 0.70 |
| SDCCAG8 | 2 | 3.6 | 669 | -0.09 | 0.21 |
| TUT1 | 3 | 4.7 | 912 | -0.09 | 0.20 |
| CEP170B | 5 | 6.2 | 1590 | -0.09 | 0.16 |
| LIN28B | 3 | 16.3 | 258 | -0.09 | 0.30 |
| PHRF1 | 2 | 2.7 | 1645 | -0.09 | 0.32 |
| PIN1 | 7 | 58.3 | 163 | -0.09 | 0.14 |
| MYL12A; MYL12B | 5 | 64.4 | 177 | -0.09 | 0.16 |
| RPS17 | 7 | 60.7 | 135 | -0.09 | 0.23 |
| TSR2 | 5 | 36.6 | 191 | -0.09 | 0.13 |
| PDLIM2 | 7 | 57.5 | 146 | -0.09 | 1.02 |
| NRD1 | 42 | 38.9 | 1219 | -0.09 | 1.33 |
| CSRP2 | 6 | 31.7 | 243 | -0.09 | 0.59 |
| CFDP1 | 3 | 37.5 | 299 | -0.09 | 0.14 |
| NUDT3 | 3 | 50 | 172 | -0.08 | 0.15 |
| BAT3; BAG6 | 11 | 16.5 | 1126 | -0.08 | 0.60 |
| FKBPL | 2 | 6 | 349 | -0.08 | 0.20 |
| NCAPH | 12 | 23 | 730 | -0.08 | 0.35 |
| FBRS | 2 | 2.2 | 980 | -0.08 | 0.16 |
| MLST8 | 2 | 25.9 | 116 | -0.08 | 0.13 |
| FKBP3 | 17 | 67.4 | 224 | -0.08 | 0.14 |
| PFN2 | 3 | 52.1 | 188 | -0.08 | 0.13 |
| CENPK | 2 | 7.9 | 239 | -0.08 | 0.20 |
| AAK1 | 9 | 34.1 | 511 | -0.08 | 0.73 |
| CLN5 | 8 | 25.1 | 358 | -0.08 | 1.43 |
| TRMT5 | 11 | 24.2 | 509 | -0.08 | 0.15 |
| MED28 | 3 | 16.8 | 179 | -0.08 | 0.16 |
| NT5C3A | 6 | 20.5 | 331 | -0.08 | 0.16 |
| OPTN | 10 | 20.3 | 577 | -0.08 | 0.17 |
| NEDD8; NEDD8-MDP1 | 2 | 60.5 | 81 | -0.08 | 0.12 |
| SUMO1 | 9 | 37.7 | 146 | -0.08 | 0.86 |
| BAIAP2 | 8 | 24.3 | 375 | -0.08 | 1.18 |
| NELFB | 2 | 3.5 | 628 | -0.08 | 0.27 |
| TACC2 | 34 | 17.2 | 2826 | -0.08 | 0.71 |
| RAB5A | 4 | 56.2 | 201 | -0.08 | 0.20 |
| CMBL | 16 | 47.8 | 245 | -0.08 | 0.14 |
| MANBA | 2 | 4.1 | 822 | -0.08 | 0.56 |
| NPEPPS | 49 | 59.6 | 915 | -0.08 | 1.60 |
| NAP1L5 | 3 | 14.3 | 182 | -0.08 | 0.13 |
| HK2 | 16 | 27 | 889 | -0.08 | 0.35 |
| CETN3 | 6 | 34.7 | 167 | -0.08 | 0.13 |
| CASP2 | 3 | 15.3 | 313 | -0.08 | 0.13 |
| SAC3D1 | 2 | 9.8 | 358 | -0.08 | 0.48 |
| MKLN1 | 30 | 53.7 | 735 | -0.08 | 0.13 |
| LRBA | 2 | 1.7 | 2575 | -0.08 | 0.16 |
| PSMD9 | 13 | 69.1 | 223 | -0.08 | 0.13 |
| IDS | 2 | 9.7 | 339 | -0.08 | 0.29 |
| GTPBP10 | 4 | 11.6 | 387 | -0.08 | 0.69 |
| RABIF | 4 | 41.5 | 123 | -0.08 | 0.14 |
| MYCBP | 7 | 59.1 | 110 | -0.08 | 0.57 |
| KNSTRN | 6 | 34 | 238 | -0.08 | 0.14 |
| FAM107B | 6 | 49.2 | 124 | -0.08 | 1.13 |
| PTMA | 8 | 40.2 | 107 | -0.07 | 0.57 |
| FPGT | 3 | 9.6 | 607 | -0.07 | 0.29 |
| C19orf25 | 4 | 51.7 | 118 | -0.07 | 0.17 |
| DTD2 | 5 | 38.7 | 168 | -0.07 | 0.21 |
| HMGN3 | 3 | 29.2 | 130 | -0.07 | 0.20 |
| SUMO2 | 2 | 47.9 | 71 | -0.07 | 0.13 |
| TTC9C | 5 | 27.5 | 171 | -0.07 | 0.13 |
| SLBP | 5 | 20.9 | 277 | -0.07 | 0.24 |
| MTFR1L | 2 | 14.9 | 195 | -0.07 | 0.57 |
| POLR2H | 10 | 79.1 | 148 | -0.07 | 1.49 |
| CRKL | 20 | 67.7 | 303 | -0.07 | 0.15 |
| PCYT1A | 9 | 33.5 | 367 | -0.07 | 0.15 |
| FAM21C | 7 | 43.7 | 1341 | -0.07 | 0.65 |
| PSMD10 | 10 | 58.8 | 226 | -0.07 | 0.11 |
| XPA | 3 | 15.2 | 264 | -0.07 | 0.38 |
| ARHGDIB | 8 | 43.3 | 201 | -0.07 | 0.15 |
| IKBKG | 17 | 45.5 | 418 | -0.07 | 0.48 |
| AKAP9 | 2 | 0.6 | 3910 | -0.07 | 0.34 |
| STXBP5 | 2 | 3.4 | 1098 | -0.07 | 0.10 |
| NUCB1 | 25 | 54.7 | 461 | -0.07 | 0.12 |
| NUCKS1 | 13 | 59.3 | 243 | -0.07 | 0.13 |
| SMAP; C11orf58 | 11 | 48.6 | 183 | -0.07 | 0.13 |
| RNF187 | 5 | 31.9 | 235 | -0.07 | 0.18 |
| PROSC | 11 | 46.5 | 275 | -0.07 | 0.12 |
| CRK | 17 | 79.9 | 304 | -0.07 | 0.14 |
| ALMS1 | 6 | 2.3 | 3859 | -0.07 | 0.92 |
| TSSC1 | 7 | 19.1 | 414 | -0.07 | 0.32 |
| LTV1 | 12 | 28.4 | 475 | -0.07 | 0.13 |
| NDUFAF5 | 2 | 12.7 | 267 | -0.07 | 0.32 |
| RBMS1; RBMS3 | 3 | 10 | 370 | -0.07 | 0.27 |
| PPIL3 | 8 | 64 | 161 | -0.07 | 0.12 |
| FAM204A | 3 | 14.2 | 219 | -0.07 | 0.43 |
| CBR1 | 15 | 73.3 | 277 | -0.07 | 0.12 |
| WRAP73 | 4 | 8 | 435 | -0.07 | 0.62 |
| EIF4EBP2 | 4 | 73.3 | 120 | -0.07 | 0.13 |
| NAGA | 5 | 16.5 | 411 | -0.07 | 0.17 |
| NUDT1 | 6 | 41.7 | 156 | -0.07 | 0.12 |
| ARFGAP2 | 7 | 16.4 | 385 | -0.07 | 0.55 |
| BAD | 5 | 57.7 | 168 | -0.07 | 0.13 |
| CXXC1 | 2 | 11.2 | 250 | -0.07 | 0.13 |
| YY1 | 7 | 17.4 | 414 | -0.07 | 0.11 |
| BANF1 | 7 | 53.9 | 89 | -0.07 | 0.14 |
| GRIPAP1 | 29 | 46.8 | 841 | -0.07 | 0.12 |
| STX1A | 10 | 43.1 | 260 | -0.07 | 0.94 |
| HEBP2 | 10 | 62 | 205 | -0.07 | 0.12 |
| TPPP | 2 | 10 | 219 | -0.07 | 0.14 |
| KIAA0101 | 7 | 68.5 | 111 | -0.06 | 0.11 |
| TRIM47 | 2 | 8.6 | 638 | -0.06 | 0.14 |
| UBXN1 | 15 | 53.8 | 290 | -0.06 | 0.55 |
| SASH1 | 2 | 3.3 | 657 | -0.06 | 0.72 |
| SDHAF3 | 2 | 22.1 | 68 | -0.06 | 0.42 |
| GTF2A1 | 8 | 18.6 | 376 | -0.06 | 0.12 |
| ZMYM3 | 5 | 18.1 | 497 | -0.06 | 0.75 |
| CBR3 | 10 | 59.2 | 277 | -0.06 | 0.10 |
| TSC22D1 | 3 | 12.2 | 1073 | -0.06 | 0.13 |
| PCBD2 | 4 | 41.2 | 119 | -0.06 | 0.12 |
| BCKDHA | 4 | 17.1 | 328 | -0.06 | 0.78 |
| SAMHD1 | 8 | 17.4 | 591 | -0.06 | 0.16 |
| FAM3C | 5 | 23.8 | 227 | -0.06 | 0.12 |
| UROS | 5 | 29.1 | 265 | -0.06 | 0.13 |
| SSB | 32 | 69.1 | 408 | -0.06 | 0.10 |
| YWHAQ | 16 | 76.3 | 245 | -0.06 | 0.11 |
| AAMP | 14 | 45.8 | 415 | -0.06 | 1.80 |
| TROVE2 | 23 | 47.4 | 538 | -0.06 | 0.12 |
| RFK | 7 | 37.4 | 155 | -0.06 | 0.12 |
| PEA15 | 2 | 72.3 | 130 | -0.06 | 0.12 |
| MRPS22 | 5 | 21.9 | 319 | -0.06 | 0.09 |
| ACTR6 | 9 | 30.6 | 376 | -0.06 | 0.57 |
| CALCOCO2 | 15 | 61.8 | 374 | -0.06 | 0.12 |
| LSM5 | 4 | 58.2 | 91 | -0.06 | 0.11 |
| AAMDC | 8 | 69.7 | 122 | -0.06 | 0.12 |
| NUDT5 | 16 | 85.8 | 232 | -0.06 | 0.37 |
| NUDCD2 | 7 | 63.1 | 157 | -0.06 | 0.11 |
| FGF1 | 5 | 49.7 | 155 | -0.06 | 0.12 |
| LSM6 | 5 | 75 | 80 | -0.06 | 0.10 |
| C12orf57 | 6 | 74.6 | 126 | -0.06 | 0.11 |
| SORBS3 | 8 | 27.1 | 329 | -0.06 | 0.12 |
| NSMCE2 | 4 | 32.6 | 187 | -0.06 | 0.64 |
| CD59 | 5 | 36.9 | 130 | -0.06 | 0.12 |
| DCAF13 | 8 | 17.4 | 597 | -0.06 | 0.30 |
| CYCS | 9 | 64.4 | 101 | -0.06 | 0.25 |
| S100A3 | 5 | 51.5 | 101 | -0.06 | 0.12 |
| SRSF2 | 6 | 36.2 | 221 | -0.06 | 0.10 |
| MRPL21 | 5 | 31.2 | 205 | -0.06 | 0.12 |
| SCAI; C9orf126 | 2 | 4.8 | 606 | -0.06 | 0.09 |
| HDGFRP3 | 6 | 42.4 | 203 | -0.06 | 0.25 |
| ALDH1L1 | 19 | 25.1 | 902 | -0.06 | 0.10 |
| TGM2 | 23 | 44.8 | 687 | -0.06 | 0.12 |
| STUB1 | 12 | 36 | 303 | -0.06 | 0.10 |
| COTL1 | 11 | 75.4 | 142 | -0.06 | 0.10 |
| TCEA1 | 15 | 52.2 | 301 | -0.06 | 0.79 |
| HLCS | 3 | 4.5 | 726 | -0.06 | 0.13 |
| RMI2 | 2 | 11.6 | 147 | -0.06 | 0.11 |
| BCL9L | 11 | 8.1 | 1462 | -0.06 | 1.40 |
| NQO2 | 10 | 60.6 | 193 | -0.06 | 0.11 |
| FABP6 | 4 | 41.4 | 128 | -0.06 | 0.10 |
| SPP1 | 3 | 17.8 | 287 | -0.06 | 0.11 |
| SH3BGRL | 10 | 85.1 | 114 | -0.06 | 0.11 |
| C1orf123 | 10 | 78.8 | 160 | -0.06 | 0.10 |
| CRACR2A | 6 | 18.7 | 395 | -0.06 | 0.10 |
| PUS1 | 14 | 38.8 | 399 | -0.06 | 0.10 |
| PALM2-AKAP2; PALM2 | 9 | 54 | 433 | -0.06 | 0.44 |
| PRDM2 | 7 | 6.3 | 1481 | -0.06 | 0.10 |
| hCG_2043597 | 7 | 65.3 | 98 | -0.06 | 0.37 |
| CCDC97 | 11 | 46.6 | 343 | -0.06 | 0.10 |
| ADAT3 | 3 | 12.8 | 367 | -0.06 | 0.20 |
| EWSR1 | 3 | 14.9 | 618 | -0.06 | 0.26 |
| NAA50 | 12 | 68.5 | 168 | -0.05 | 0.57 |
| RAD23B | 14 | 58.9 | 409 | -0.05 | 0.09 |
| DDT; DDTL | 12 | 95.8 | 118 | -0.05 | 0.08 |
| ARF5 | 2 | 44.4 | 180 | -0.05 | 0.12 |
| PKIG | 4 | 92.1 | 76 | -0.05 | 0.09 |
| PPP2R4 | 11 | 50.2 | 329 | -0.05 | 2.11 |
| DMD | 4 | 0.9 | 3680 | -0.05 | 0.18 |
| FAM21A | 7 | 44.1 | 1253 | -0.05 | 0.54 |
| PIR | 13 | 55.5 | 290 | -0.05 | 0.09 |
| MACF1 | 2 | 23.5 | 1375 | -0.05 | 0.18 |
| BIRC2 | 3 | 5.9 | 597 | -0.05 | 0.42 |
| NRGN; GAP43 | 2 | 48.7 | 78 | -0.05 | 0.09 |
| HYI | 4 | 27.2 | 272 | -0.05 | 0.54 |
| WDR46 | 2 | 7.2 | 610 | -0.05 | 0.09 |
| MIER1 | 12 | 33.8 | 485 | -0.05 | 0.10 |
| EIF4B | 27 | 54.9 | 616 | -0.05 | 0.45 |
| NFYB | 3 | 19.1 | 131 | -0.05 | 0.36 |
| PAGE5 | 9 | 93.6 | 110 | -0.05 | 0.08 |
| DCP1A | 3 | 7.4 | 544 | -0.05 | 0.11 |
| TOLLIP | 9 | 42.7 | 274 | -0.05 | 0.09 |
| ST13; ST13P5; ST13P4 | 18 | 50.9 | 369 | -0.05 | 0.10 |
| CASP3 | 9 | 52.3 | 277 | -0.05 | 0.09 |
| ZFYVE19 | 9 | 30.1 | 448 | -0.05 | 0.10 |
| POP7 | 3 | 27 | 137 | -0.05 | 0.38 |
| MBLAC1 | 4 | 28.2 | 266 | -0.05 | 0.37 |
| MTRF1L | 5 | 14.7 | 380 | -0.05 | 0.10 |
| CREB1 | 4 | 25.8 | 341 | -0.05 | 0.09 |
| FBXO3 | 8 | 23.5 | 430 | -0.05 | 0.79 |
| ACTN4 | 2 | 82.2 | 342 | -0.05 | 0.10 |
| C18orf25; ARKL1 | 9 | 42.1 | 342 | -0.05 | 0.08 |
| MRFAP1 | 4 | 42.5 | 127 | -0.05 | 0.08 |
| STMN1 | 9 | 57 | 149 | -0.05 | 0.10 |
| APEX1 | 19 | 67.3 | 318 | -0.05 | 0.08 |
| THYN1 | 9 | 32.9 | 225 | -0.05 | 0.08 |
| EEF1B2 | 16 | 90.7 | 225 | -0.05 | 0.09 |
| CPA4 | 16 | 58.7 | 421 | -0.05 | 0.08 |
| PHLDA2 | 2 | 9.9 | 152 | -0.05 | 0.11 |
| BOLA1 | 6 | 59.9 | 137 | -0.05 | 0.09 |
| SLC38A10 | 3 | 8 | 1119 | -0.05 | 0.10 |
| ATF1 | 4 | 23.2 | 271 | -0.05 | 0.09 |
| RBPJ | 12 | 34.8 | 465 | -0.05 | 0.08 |
| HIRIP3 | 13 | 22.3 | 556 | -0.05 | 0.10 |
| KBTBD4 | 5 | 11.2 | 543 | -0.05 | 0.29 |
| SH3BGRL3 | 6 | 42 | 88 | -0.05 | 0.10 |
| GON4L | 2 | 2.3 | 970 | -0.05 | 0.25 |
| SCRN2 | 5 | 15 | 433 | -0.05 | 0.08 |
| PDF | 2 | 17.3 | 243 | -0.05 | 0.10 |
| VPS29 | 9 | 51.4 | 214 | -0.05 | 0.40 |
| PPM1A | 11 | 41.9 | 382 | -0.05 | 0.09 |
| CCDC186 | 16 | 24.4 | 898 | -0.05 | 0.10 |
| ESD | 15 | 78 | 282 | -0.05 | 0.11 |
| PLEKHA5 | 13 | 17.5 | 874 | -0.05 | 0.48 |
| MCFD2 | 3 | 69.1 | 94 | -0.05 | 0.07 |
| RPIA | 17 | 72.7 | 311 | -0.05 | 0.09 |
| IVD | 8 | 23.7 | 426 | -0.05 | 0.26 |
| TPT1 | 12 | 78.2 | 188 | -0.05 | 0.08 |
| SHMT1 | 16 | 51.4 | 444 | -0.05 | 0.09 |
| PDXK | 9 | 43.8 | 272 | -0.05 | 0.32 |
| SRP9 | 8 | 72.1 | 86 | -0.05 | 0.07 |
| RP9 | 8 | 31.7 | 221 | -0.05 | 0.08 |
| MPST | 14 | 53.9 | 297 | -0.05 | 0.07 |
| WIPF2 | 9 | 21.6 | 440 | -0.05 | 0.08 |
| ANXA1 | 27 | 76 | 346 | -0.05 | 0.09 |
| PIN4 | 9 | 67.2 | 131 | -0.05 | 0.08 |
| RASAL2 | 3 | 4.4 | 1139 | -0.05 | 0.07 |
| Em:AP000351.3; GSTT2; GSTT2B | 8 | 44.3 | 230 | -0.05 | 0.09 |
| DNAJC8 | 20 | 66.8 | 253 | -0.05 | 0.08 |
| DYRK1A | 5 | 11.3 | 529 | -0.05 | 0.07 |
| PSMG1 | 14 | 63.9 | 288 | -0.05 | 0.09 |
| SMARCE1 | 15 | 50.9 | 393 | -0.05 | 0.37 |
| SLC9A3R2 | 2 | 27.3 | 326 | -0.05 | 0.07 |
| CBX3 | 8 | 61.7 | 183 | -0.05 | 0.08 |
| NMT1 | 13 | 43.1 | 496 | -0.05 | 0.08 |
| WDR44 | 30 | 42.5 | 905 | -0.05 | 0.08 |
| TXNDC17 | 10 | 84.6 | 123 | -0.05 | 0.08 |
| SBSN | 2 | 5.4 | 590 | -0.05 | 0.08 |
| EML3 | 13 | 19.8 | 897 | -0.05 | 0.46 |
| RPS28 | 3 | 36.2 | 69 | -0.05 | 0.07 |
| PRMT1 | 2 | 73.7 | 353 | -0.05 | 0.07 |
| MAP4 | 39 | 55.8 | 2297 | -0.05 | 0.36 |
| MAP1A | 12 | 6.1 | 3041 | -0.04 | 0.35 |
| GPALPP1 | 11 | 29.6 | 338 | -0.04 | 0.31 |
| HNRNPUL1 | 19 | 35.9 | 766 | -0.04 | 0.40 |
| MSRA | 9 | 62.4 | 213 | -0.04 | 0.07 |
| PREPL | 23 | 48.3 | 638 | -0.04 | 0.07 |
| UBE2B | 3 | 58.6 | 152 | -0.04 | 0.08 |
| EEFSEC | 3 | 6 | 436 | -0.04 | 0.19 |
| CFAP45 | 7 | 16.5 | 466 | -0.04 | 0.08 |
| SGTB | 8 | 59.5 | 195 | -0.04 | 0.22 |
| BRMS1L | 2 | 14.9 | 215 | -0.04 | 0.07 |
| WNK1 | 15 | 9 | 2134 | -0.04 | 0.49 |
| LCMT1 | 10 | 33.2 | 334 | -0.04 | 0.08 |
| C5orf22 | 10 | 38.5 | 442 | -0.04 | 0.08 |
| TBCA | 11 | 78.6 | 84 | -0.04 | 0.32 |
| NENF | 9 | 62.8 | 172 | -0.04 | 0.07 |
| WDR6 | 11 | 13.5 | 1151 | -0.04 | 0.64 |
| ANKRD40 | 7 | 25.5 | 368 | -0.04 | 0.07 |
| FSCN1 | 38 | 76.3 | 493 | -0.04 | 0.07 |
| GEMIN6 | 6 | 52.1 | 167 | -0.04 | 0.07 |
| RPAIN | 2 | 10.1 | 148 | -0.04 | 0.35 |
| CCDC91 | 13 | 32.1 | 411 | -0.04 | 0.07 |
| NAV3 | 2 | 2.7 | 679 | -0.04 | 0.42 |
| HDHD1 | 7 | 49.2 | 191 | -0.04 | 0.68 |
| HMCES | 3 | 11.9 | 312 | -0.04 | 0.09 |
| AARS | 42 | 54.6 | 968 | -0.04 | 0.08 |
| HEXIM1 | 15 | 64.1 | 359 | -0.04 | 0.07 |
| TRIP12 | 4 | 3 | 1992 | -0.04 | 0.08 |
| UBE2A | 3 | 57.2 | 152 | -0.04 | 0.07 |
| NASP | 23 | 87.3 | 788 | -0.04 | 0.07 |
| SEPT11 | 9 | 49.6 | 425 | -0.04 | 0.23 |
| INPP1 | 2 | 20.3 | 143 | -0.04 | 0.17 |
| DNAJC24; ZCSL3 | 5 | 60.7 | 84 | -0.04 | 0.07 |
| ALDOC | 9 | 85.4 | 364 | -0.04 | 0.08 |
| PEBP1 | 12 | 79.7 | 187 | -0.04 | 0.08 |
| WDR20 | 10 | 19.8 | 612 | -0.04 | 0.35 |
| SDF2 | 3 | 29.4 | 211 | -0.04 | 0.07 |
| PCNP | 12 | 58.4 | 178 | -0.04 | 0.07 |
| NDE1 | 12 | 55.8 | 335 | -0.04 | 0.09 |
| FAM188A | 9 | 29.2 | 445 | -0.04 | 0.06 |
| MAML1 | 3 | 4.6 | 1016 | -0.04 | 0.08 |
| PAGE1 | 3 | 28.8 | 146 | -0.04 | 0.06 |
| DBNL | 2 | 55.3 | 430 | -0.04 | 0.07 |
| GRB2 | 19 | 84.8 | 217 | -0.04 | 0.07 |
| S100A2 | 3 | 25.5 | 98 | -0.04 | 0.08 |
| MRPL14 | 2 | 12.4 | 145 | -0.04 | 0.08 |
| PPP1R2; PPP1R2P3 | 11 | 84.9 | 205 | -0.04 | 0.07 |
| S100A11 | 6 | 55.2 | 105 | -0.04 | 0.06 |
| OGFR | 17 | 44.7 | 657 | -0.04 | 0.08 |
| ANXA5 | 27 | 80.6 | 320 | -0.04 | 0.07 |
| SEC24B | 5 | 6.9 | 1233 | -0.04 | 0.06 |
| PHF23 | 3 | 20.3 | 158 | -0.04 | 0.20 |
| COX6B1 | 9 | 83.7 | 86 | -0.04 | 0.06 |
| PTPN2 | 3 | 14 | 386 | -0.04 | 0.07 |
| CFL1 | 2 | 86.1 | 166 | -0.04 | 0.06 |
| ACP1 | 2 | 71.5 | 158 | -0.04 | 0.08 |
| HAGH | 13 | 55.5 | 308 | -0.04 | 0.07 |
| AK1 | 15 | 68.1 | 210 | -0.04 | 0.07 |
| GNS | 13 | 31.5 | 552 | -0.04 | 0.06 |
| TALDO1 | 22 | 52.5 | 337 | -0.04 | 0.06 |
| SLC4A1AP | 30 | 55.8 | 742 | -0.04 | 0.56 |
| LRRFIP2 | 15 | 43.4 | 424 | -0.04 | 0.07 |
| ATOX1 | 7 | 80.9 | 68 | -0.04 | 0.07 |
| NUDT9 | 13 | 45 | 300 | -0.04 | 0.07 |
| SSU72 | 7 | 39.2 | 194 | -0.04 | 0.06 |
| NOTCH2 | 10 | 7.6 | 2471 | -0.04 | 0.07 |
| LASP1 | 20 | 83.9 | 261 | -0.04 | 0.08 |
| SCRN1 | 22 | 68.1 | 414 | -0.04 | 0.07 |
| ZRANB2 | 19 | 44.7 | 320 | -0.04 | 0.06 |
| CSTA | 7 | 75.5 | 98 | -0.04 | 0.09 |
| CNPY4 | 8 | 39.1 | 248 | -0.04 | 0.07 |
| GSTM3 | 3 | 19.8 | 212 | -0.04 | 0.23 |
| INF2 | 5 | 8.5 | 1240 | -0.04 | 0.07 |
| DPP3 | 37 | 64.3 | 737 | -0.04 | 0.06 |
| PTGES2 | 2 | 9.7 | 186 | -0.04 | 0.26 |
| AACS | 4 | 6.1 | 672 | -0.04 | 0.06 |
| MSN | 35 | 78 | 577 | -0.04 | 0.06 |
| NMNAT1 | 4 | 15.4 | 279 | -0.04 | 0.06 |
| ERC1 | 27 | 34.6 | 1120 | -0.04 | 0.07 |
| BLOC1S2 | 7 | 46.5 | 142 | -0.04 | 0.08 |
| CFL2 | 13 | 89.8 | 166 | -0.04 | 0.07 |
| MEA1 | 2 | 16.2 | 185 | -0.04 | 0.06 |
| NSFL1C | 30 | 75.4 | 370 | -0.04 | 0.06 |
| TCEB2 | 15 | 97.5 | 118 | -0.04 | 0.06 |
| CHD1 | 6 | 3.2 | 1709 | -0.04 | 0.06 |
| OBSL1 | 2 | 2.8 | 612 | -0.04 | 0.19 |
| MOCS2 | 10 | 64.4 | 188 | -0.04 | 0.07 |
| IMPDH1 | 10 | 27.7 | 513 | -0.04 | 0.60 |
| CRIP2 | 7 | 48.4 | 192 | -0.04 | 0.05 |
| SLIRP | 6 | 63.6 | 107 | -0.04 | 0.07 |
| MYL6B | 10 | 59.1 | 208 | -0.04 | 0.06 |
| DIAPH3 | 2 | 1.5 | 1123 | -0.04 | 0.06 |
| DLGAP4 | 15 | 41.9 | 453 | -0.04 | 0.06 |
| ING3 | 4 | 15.4 | 403 | -0.04 | 0.14 |
| PDCD5 | 14 | 72 | 125 | -0.04 | 0.07 |
| POLR2J3; POLR2J; POLR2J2 | 3 | 27 | 115 | -0.04 | 0.34 |
| EIF4EBP1 | 9 | 80.5 | 118 | -0.04 | 0.06 |
| DCTN6 | 8 | 56.8 | 190 | -0.04 | 0.06 |
| ZFP64 | 3 | 10.6 | 426 | -0.04 | 0.07 |
| RPS24 | 6 | 47.3 | 131 | -0.04 | 0.18 |
| FBXO45 | 2 | 16.8 | 286 | -0.04 | 0.08 |
| CDA | 6 | 67.8 | 146 | -0.04 | 0.06 |
| SCYL2 | 3 | 5.1 | 681 | -0.04 | 0.32 |
| GLOD4 | 20 | 72.8 | 298 | -0.04 | 0.06 |
| NUP37 | 11 | 39.3 | 326 | -0.04 | 0.06 |
| CENPC | 2 | 4.7 | 728 | -0.04 | 0.06 |
| RNF4 | 4 | 41.5 | 188 | -0.04 | 0.24 |
| TNRC6C | 2 | 1.2 | 1944 | -0.04 | 0.23 |
| KCTD5 | 7 | 56 | 234 | -0.04 | 0.06 |
| PA2G4 | 29 | 81.5 | 394 | -0.03 | 0.05 |
| MYO1C | 8 | 12.3 | 1039 | -0.03 | 0.28 |
| SYNJ2BP-COX16; SYNJ2BP | 7 | 60.5 | 152 | -0.03 | 0.24 |
| ANP32B | 8 | 35.5 | 251 | -0.03 | 0.06 |
| NAGLU | 5 | 8.7 | 743 | -0.03 | 0.06 |
| C15orf40 | 4 | 44.4 | 153 | -0.03 | 0.06 |
| TIPIN | 4 | 15.6 | 294 | -0.03 | 0.07 |
| NR2C2AP | 6 | 71.2 | 139 | -0.03 | 0.06 |
| PPIA | 6 | 87.3 | 165 | -0.03 | 0.05 |
| PEX5 | 2 | 9.2 | 272 | -0.03 | 0.11 |
| MEMO1 | 15 | 76.8 | 297 | -0.03 | 0.05 |
| PSMA2 | 15 | 71.4 | 234 | -0.03 | 0.16 |
| KCNAB2 | 2 | 5.7 | 300 | -0.03 | 0.06 |
| DHX36 | 26 | 35 | 994 | -0.03 | 0.05 |
| CLIC1 | 17 | 88.8 | 241 | -0.03 | 0.06 |
| KIAA1671 | 4 | 2.9 | 1806 | -0.03 | 0.06 |
| RNF169 | 3 | 4 | 708 | -0.03 | 0.07 |
| RNASEH2C | 4 | 46.2 | 158 | -0.03 | 0.08 |

**Table S3.** qPCR and ChIP primer sequences.

| *Name* | *Method* | *Forward* | *Reverse* |
| --- | --- | --- | --- |
| BCL6 | qPCR | CTGCAGATGGAGCATGTTGT | TCTTCACGAGGAGGCTTGAT |
| CIS | qPCR | CTGCTGTGCATAGCCAAGAC | GTGCCTTCTGGCATCTTCTG |
| DHFR | qPCR | CCTGGTTCTCCATTCCTGAG | TGAGCTCCTTGTGGAGGTTC |
| GAPDH | qPCR | AATCCCATCACCATCTTCCA | TGGACTCCACGACGTACTCA |
| ICAM1 | qPCR | CGGCCAGCTTATACACAAGA | CATTGGAGTCTGCTGGGAAT |
| JUNB | qPCR | AAATGGAACAGCCCTTCT | TGTAGAGAGAGGCCACCA |
| MCL1 | qPCR | GAGACCTTACGACGGGTT | TTTGATGTCCAGTTTCCG |
| SOCS3 | qPCR | TCAAGACCTTCAGCTCCAAG | TGACGCTGAGCGTGAAGAAG |
| STAT3 | qPCR | ACCGGCGTCCAGTTCACTACT | CCGGGATCCTCTGAGAGCTGC |
| SURVIVIN | qPCR | GGACCACCGCATCTCTACAT | GTCTGGCTCGTTCTCAGTGG |
| TNFRSF1A | qPCR | TGCCATGCAGGTTTCTTTCT | CACAACTTCGTGCACTCCAG |
| UGCG | qPCR | TTCGTCCTCTTCTTGGTGCT | AGACACCTGGGAGCTTGCTA |
| BCL6 | ChIP | CGGCAGCAACAGCAATAATC | GGAGAGCTGACACCAAGTCC |
| ICAM1 | ChIP | GTCCGGAAATAACTGCAGCA | GCTGAGGTTGCAACTCTGAG |
| STAT3 | ChIP | CCTGATACAGCTCCCTCCTG | GATTCCCGCGTGGTAAGAG |
| TNFRSF1A | ChIP | GAAGGGAAGTCTCCCTCGAC | CACGGAATGGGTTTAGCTGT |
| UGCG | ChIP | GCAGGGTTCTCTCTTGGAGAT | GCCATGATCCTCAAACCATT |
